# Erythropoietin modulates bone marrow stromal cell differentiation

**DOI:** 10.1038/s41413-019-0060-0

**Published:** 2019-07-25

**Authors:** Sukanya Suresh, Luis Fernandez de Castro, Soumyadeep Dey, Pamela G. Robey, Constance Tom Noguchi

**Affiliations:** 1 Molecular Medicine Branch, National Institute of Diabetes and Digestive and Kidney Diseases, National Institutes of Health, Bethesda, MD 20892 USA; 2 Skeletal Biology Section, National Institute of Dental and Craniofacial Research, National Institutes of Health, Bethesda, MD 20892 USA

**Keywords:** Bone, Fat metabolism

## Abstract

Erythropoietin is essential for bone marrow erythropoiesis and erythropoietin receptor on non-erythroid cells including bone marrow stromal cells suggests systemic effects of erythropoietin. Tg6 mice with chronic erythropoietin overexpression have a high hematocrit, reduced trabecular and cortical bone and bone marrow adipocytes, and decreased bone morphogenic protein 2 driven ectopic bone and adipocyte formation. Erythropoietin treatment (1 200 IU·kg^–1^) for 10 days similarly exhibit increased hematocrit, reduced bone and bone marrow adipocytes without increased osteoclasts, and reduced bone morphogenic protein signaling in the bone marrow. Interestingly, endogenous erythropoietin is required for normal differentiation of bone marrow stromal cells to osteoblasts and bone marrow adipocytes. ΔEpoR_E_ mice with erythroid restricted erythropoietin receptor exhibit reduced trabecular bone, increased bone marrow adipocytes, and decreased bone morphogenic protein 2 ectopic bone formation. Erythropoietin treated ΔEpoR_E_ mice achieved hematocrit similar to wild-type mice without reduced bone, suggesting that bone reduction with erythropoietin treatment is associated with non-erythropoietic erythropoietin response. Bone marrow stromal cells from wild-type, Tg6, and ΔEpoR_E_-mice were transplanted into immunodeficient mice to assess development into a bone/marrow organ. Like endogenous bone formation, Tg6 bone marrow cells exhibited reduced differentiation to bone and adipocytes indicating that high erythropoietin inhibits osteogenesis and adipogenesis, while ΔEpoR_E_ bone marrow cells formed ectopic bones with reduced trabecular regions and increased adipocytes, indicating that loss of erythropoietin signaling favors adipogenesis at the expense of osteogenesis. In summary, endogenous erythropoietin signaling regulates bone marrow stromal cell fate and aberrant erythropoietin levels result in their impaired differentiation.

## Introduction

Erythropoietin (EPO), a cytokine produced in fetal liver and adult kidneys, is required for production of red blood cells. EPO binds to its receptor, EPOR, expressed on erythroid progenitors and promotes survival, proliferation, and differentiation.^1^ Mice with targeted deletion of *Epo* or *Epor* die in utero of severe anemia.^2,3^ Recombinant human EPO is clinically used to treat anemia resulting from chronic kidney disease and chemotherapy.^4^ Functional EPOR is present in endothelial cells,^5^ neurons,^6^ skeletal muscle progenitor cells,^7^ adipocytes,^8^ and islets^9^ suggesting that endogenous EPO signaling exerts systemic regulation and that EPO administered in patients could interact with EPOR in non-erythroid cells with implications beyond erythropoiesis. To understand the full spectrum of clinical utility of EPO, studies to elucidate EPO response in non-erythroid cells are required.

Bone marrow contains two distinct types of stem cells: hematopoietic stem cells that give rise to all types of blood cells and skeletal stem cells (SSCs), a subset of bone marrow stromal cells (BMSCs) that differentiate into chondrocytes, osteoblasts, hematopoiesis supporting stroma and adipocytes.^10^ Lineage commitment and differentiation of SSCs/BMSCs depends on interplay of transcription factors and signaling molecules. Among several transcription factors regulating SSC/BMSC differentiation, PPAR-γ, CEBP-α, and CEBP-β enhance adipogenesis, whereas RUNX2 and OSTERIX are important for osteogenesis. Several cytokines present in the bone marrow niche also preferentially regulate SSC/BMSC differentiation.^11^ Impaired SSC/BMSC differentiation results in imbalance of adipocyte and osteoblast differentiation in marrow and clinical studies show expansion of marrow fat is associated with reduced bone density.^12,13^ Enhanced marrow adipogenesis and bone fragility are also observed in osteoporosis,^14^ obesity,^15^ and diabetes.^16^

Bone is metabolically active and undergoes continuous remodeling processes whereby osteoclasts of hematopoietic origin digest old bone and osteoblasts of BMSC origin lay down new bone matrix.^17^ EPO has been shown to promote the differentiation of these cells in in vitro culture conditions,^18^ although lineage tracing using *Epor*-Cre knock-in mice showed labeling only in cells of premegakaryocyte/erythroid origin in the bone marrow microenvironment.^19^ In fracture healing models, EPO demonstrated a beneficial effect on bone^20,21^ and accelerated wound healing, and supraphysiologic EPO promoted bone growth in vertebrae of young and old mice.^18^ Exogenous EPO treatment in animal models induced low bone mass in long bones,^22^ indicating that skeletal response to EPO depends on location of the bone, EPO dose, and extent of bone injury. Here, we show that EPOR is expressed on BMSCs and that high EPO inhibits osteogenesis and adipogenesis using transgenic mice with EPO overexpression (Tg6)^23^ that exhibit increased hematocrit, reduced bone, and reduction in bone marrow adipocytes. We also show that endogenous EPO regulates bone homeostasis and marrow adipogenesis using transgenic mouse with *Epor* expression restricted to erythroid cells (ΔEpoR_E_)^24^ in which lack of EPOR signaling in SSCs/BMSCs disrupts their differentiation to promote adipogenesis and decrease osteogenesis.

## Results

### Chronic elevated EPO levels reduce*s* bones in mice

**Fig. 1:**
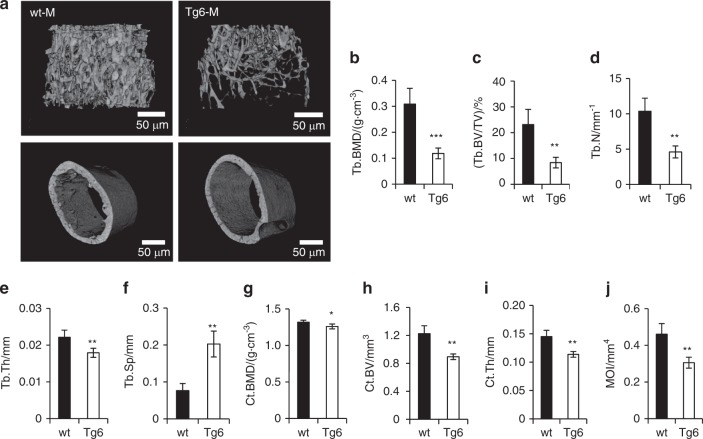
Bone features of Tg6 mice overexpressing EPO. **a** Micro-CT generated 3D images of trabecular (top) and cortical bone (bottom) of the femurs of 11-week-old wild-type (wt; left) and Tg6 mice (right). **b–f** Quantification of trabecular parameters including trabecular bone mineral density (BMD), bone volume/total volume (BV/TV), number (N), thickness (Th), and spacing (Sp) of trabeculae. **g–j** Quantification of cortical BMD, BV, thickness, and moment of inertia (MOI) (*n* = 4/group, *P < 0.05, **P < 0.01, ***P < 0.001)

To analyze the effect of elevated EPO on bone architecture, we used the transgenic Tg6 mice with overexpression of human EPO. The Tg6 mice have mouse EPO levels similar to their littermate controls but have high levels of human EPO in their circulation (Supplementary Fig. 1b), similar to earlier reports.^23^ Micro-Ct scans (Fig. 1a) showed 61% reduced trabecular bone mineral density (BMD) (Fig. 1b) and 63% reduced trabecular bone volume/tissue volume (BV/TV) (Fig. 1c) in 11-week-old male Tg6 mice compared with littermate controls. Tg6 mice also had reduced trabecular number (Fig. 1d), thickness (Fig. 1e), and increased trabecular spacing (Fig. 1f). Analysis of cortical bone parameters of Tg6 mice showed reduced cortical BMD, (Fig. 1g) BV (Fig. 1h), thickness (Fig. 1i), and moment of inertia (Fig. 1j). We compared femur length and body weight of male and female Tg6 mice and found that only male Tg6 mice had shorter femurs (Supplementary Fig. 2a) and lower body mass (Supplementary Fig. 2b). Female Tg6 mice were not different in these parameters compared with female littermate controls.

### Mice with erythroid-restricted *Epor* expression have reduced bone histomorphometric parameters

**Fig. 2:**
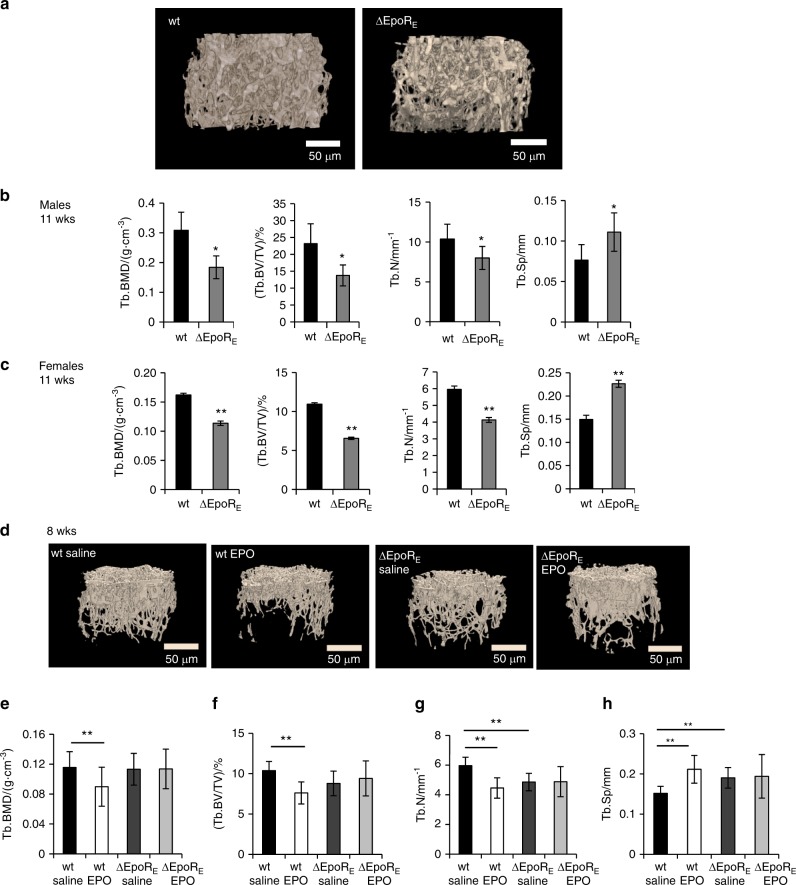
Bone features of ΔEpoR_E_ mice with lack of EPOR signaling in non-erythroid cells. **a** Micro-CT generated images of trabecular bone of 11-week-old male wild-type (wt) and ΔEpoR_E_mice. **b–c** Femurs of 11-week-old male and female ΔEpoR_E_ mice and age matched wt mice were analyzed using micro-CT. Quantitation of trabecular BMD, BV/TV, number, and spacing of trabecular bone of male (**b**) and female ΔEpoR_E_ mice (**c**) and controls (*n* = 4/group). **d**–**h** EPO induced changes in bones of 8-week-old wild-type (wt) and ΔEpoR_E_ mice with *Epor* deletion in non-erythroid cells. **d** 3D images of trabecular bones of wt and ΔEpoR_E_ mice receiving 1 200 IU·kg^–1^ of EPO for 10 days. Quantification of trabecular parameters including trabecular BMD (**e**), BV/TV (**f**), trabecular number (**g**), and trabecular spacing (**h**). (*n* = 5/group, *P < 0.05, **P < 0.01, ***P < 0.001)

We used transgenic ∆EpoR_E_-mouse model with *Epor* expression restricted to erythroid cells to assess the role of endogenous EPO signaling in regulation of osteogenic cells. Consistent with the previous report,^24^ we observed similar mouse EPO levels in the serum of ∆EpoR_E_ mice and wild-type controls (Supplementary Fig. 1a). Micro-Ct measurements of 11-week-old male ∆EpoR_E_ mice (Fig. 2a) showed 40% reduced trabecular BMD, 40.5% reduced trabecular BV/TV, 22.8% reduced trabecular number, and 45.2% increase in trabecular spacing (Fig. 2b). Female ∆EpoR_E_ mice also exhibited reduced trabecular bone with 29.8% reduced BMD, 40% reduced trabecular BV/TV, 30.6% fewer trabeculae, and 51.2% increase in trabecular spacing compared to wild-type female mice (Fig. 2c). There was no significant difference in cortical bone of both male and female ∆EpoR_E_ mice compared with control mice (Supplementary Fig. 2e, f). Femoral length was also not different in ∆EpoR_E_ mice compared with wild-type mice (Supplementary Fig. 2c). Male and female ∆EpoR_E_ mice had increased body mass (Supplementary Fig. 2d), consistent with previous reports.^8^ To determine if reduced bone mass seen with ∆EpoR_E_ mice is due to increased body weight, age and weight-matched female wild-type mice were compared with female ∆EpoR_E_ mice. In female ∆EpoR_E_ mice, reduced trabecular bone was extensive (Supplementary Figs. 2g, h, i) and cortical BV was lower (Supplementary Fig. 2j) compared with age and weight-matched control mice.·

### Elevated EPO does not reduce bone parameters in mice with *Epor* deletion in non-erythroid cells

To determine whether reduction in bone with high EPO is associated with EPO-stimulated erythropoiesis or mediated by non-erythroid cells, we treated young wild-type and ∆EpoR_E_ mice (8-week-old females) with EPO (1 200 IU·kg^–1^) for 10 days (Fig. 2d). EPO treatment increased hematocrit similarly in wild-type and ∆EpoR_E_ mice from 49% ± 1.36% and 49.5% ± 1.36% to 68.4% ± 2.23% and 70.4% ± 1.51%, respectively. EPO treatment reduced trabecular BMD (−22%) (Fig. 2e), BV/TV (−26.6%) (Fig. 2f), trabecular number (−25%) (Fig. 2g), and increased trabecular spacing (39.5%) (Fig. 2h) in wild-type mice, but did not induce any changes in the bones of ∆EpoR_E_ mice. Body weight of wild-type and ∆EpoR_E_-mice with and without EPO treatment was similar (data not shown), excluding the influence of body weight-associated changes in bone parameters. Compared with wild-type control mice, ∆EpoR_E_ mice not treated with EPO showed reduced trabecular number (−18.5%) (Fig. 2g) and increased trabecular spacing (25.5%) (Fig. 2h).

### Bone marrow adipocytes are responsive to EPO

**Fig. 3:**
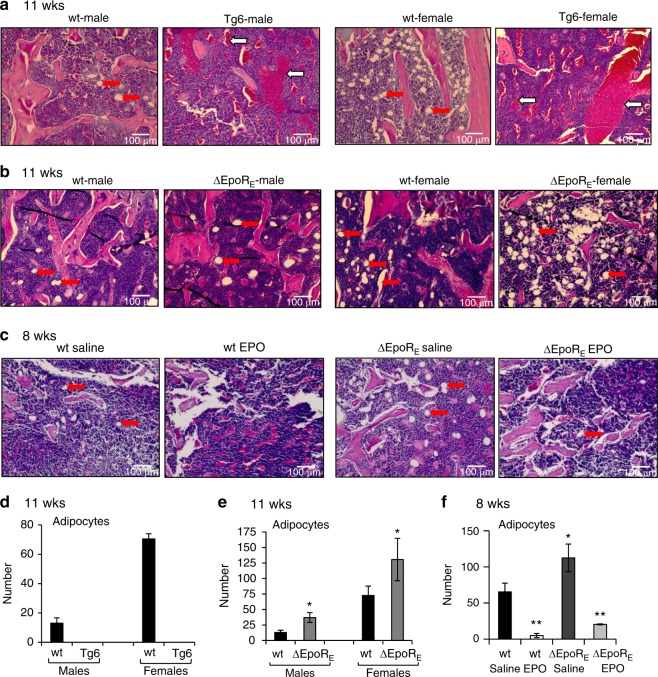
Histology analysis by H&E staining of femurs sections of Tg6- and ΔEpoR_E_-mice. **a** Femurs of 11-week-old male and female Tg6 mice and controls. Red arrows indicate marrow adipocytes, which appear as white circles in the marrow, pink colored regions are bones and blue regions are the marrow (BM). White arrows in Tg6 histology images show sinusoids (*n* = 4/group). **b** Femurs of 11-week-old male and female ΔEpoR_E_ mice and controls (*n* = 4/group). **c** Femur sections of 8-week-old wt and ΔEpoR_E_ mice receiving 1 200 IU·kg^–1^ of EPO for ten days. **d** Number of marrow adipocytes in femurs of male and female Tg6 mice (*n* = 5/group). **e** Number of marrow adipocytes in the femurs of wild-type and ΔEpoR_E_ mice. **f** Number of adipocytes in the femurs of wild-type and ΔEpoR_E_mice receiving 1 200 IU·kg^–1^ of EPO or saline treatment for ten days. *P < 0.05, **P < 0.01, ***P < 0.001

To assess EPO influence on the bone marrow fat depot, we analyzed H&E stained femur sections of Tg6 mice (11 weeks old) and their littermate controls (Fig. 3a, d). Both male and female Tg6 mice showed extensive loss of marrow adipocytes. In contrast, analysis of femur sections of male and female ∆EpoR_E_ mice showed increased marrow adipocytes (Fig. 3b, e). In both wild-type and ∆EpoR_E_ groups, female mice had significantly more marrow adipocytes than age-matched male mice. We next investigated changes in bone marrow adipocytes with exogenous administration of EPO. Both wild-type and ∆EpoR_E_ mice (8 weeks old, female) treated with EPO showed reduced bone marrow adipocytes compared with saline treatment (Fig. 3c, f).

### Effect of EPO signaling on osteogenic and osteoclast differentiation in vitro

**Fig. 4:**
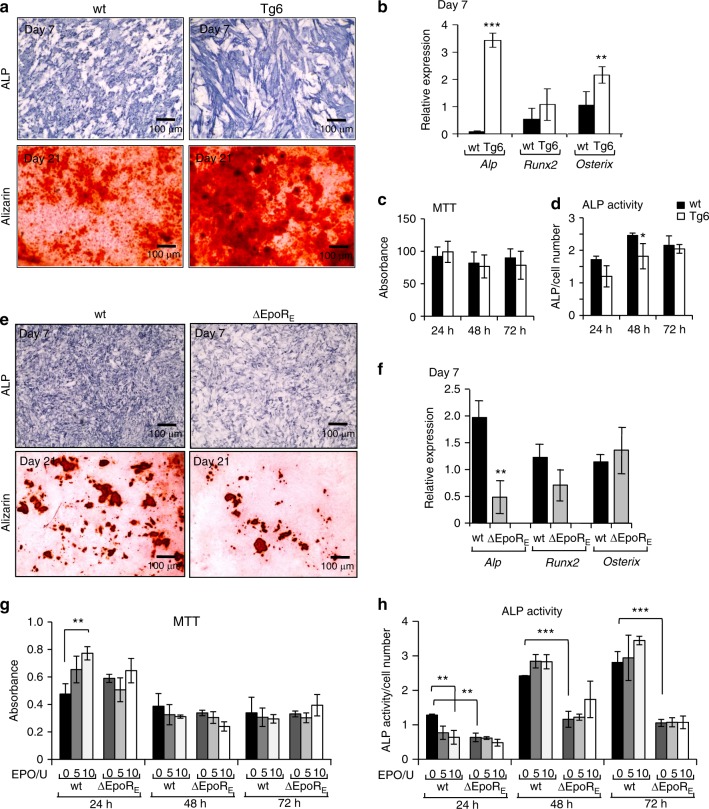
Osteogenic cultures of Tg6- and ΔEpoR_E_-mice. **a** Calvarial osteoprogenitors isolated from wt and Tg6-mice were cultured in osteogenic medium and stained for ALP expression on day 7 and mineralization deposits by alizarin red staining on day 21. **b** Relative expression of *Alp*, *Runx2* and *Osterix* in osteogenic cultures on day 7 determined by real-time PCR. **c** Cell proliferation of calvarial osteoblasts was determined using MTT assay at 24, 48, and 72 h of culture in osteogenic medium and data expressed as absorbance values. **d** ALP activity was measured using a colorimetric assay and expressed as absorbance normalized to the cell number determined by MTT assay. **e** Calvarial osteoprogenitors isolated from wt and ΔEpoR_E_-mice cultured in osteogenic medium and stained for ALP (day 7) and alizarin red (day 21). **f** Relative expression of *Alp*, *Runx2* and *Osterix* mRNA levels from primary calvarial osteogenic cells (day 7) determined by real-time PCR. **g**–**h** Calvarial osteogenic cells from wt and ΔEpoR_E_-mice treated with 5 U and 10 U of EPO and measured for their cell proliferation rate by MTT assay (**g**) and ALP activity of the cells were measured using colorimetric assays, absorbance was normalized to the cell number (**h**). (*n* = 4/group, **P < 0.01, ***P < 0.001)

We performed in vitro differentiation assays to determine if chronically elevated EPO has a direct effect on osteogenic cells and osteoclasts. Long-term cultures of calvarial osteogenic cells in osteogenic medium (Fig. 4a) showed that Tg6-osteogenic cells having increased mineralization potential along with increased *Alp* and *Osterix* expression with no significant changes in *Runx2* (Fig. 4b). Tg6-osteogenic cells also expressed human EPO, although *Epor* levels were similar to wild-type osteogenic cells (Supplementary Fig. 3a, b). High EPO levels in these cells did not affect their viability (Fig. 4c). Except for a transient decrease in ALP activity measured at 48 h in osteogenic cultures from Tg6 mice, no significant differences in ALP activity were observed (Fig. 4d).

Primary calvarial osteogenic cells of ∆EpoR_E_ mice cultured in osteogenic medium showed lower differentiation and mineralization as identified by ALP and alizarin red staining, respectively (Fig. 4e). Gene expression analysis showed less *Alp* expression, but no changes in *Runx2* and *Osterix* levels in ∆EpoR_E_-osteogenic cells cultured for 7 days in osteogenic medium (Fig. 4f). Addition of EPO to in vitro cultures of wild-type osteogenic cells resulted in a transient increase in viability along with reduction in their differentiation potential at 24 h as evidenced by decreased ALP activity (Fig. 4g, h). However, long-term calvarial osteogenic cultures of wild-type mice with EPO addition did not show any effect in their differentiation and mineralization ability (data not shown). The osteogenic cells from ∆EpoR_E_ mice had a reduced differentiation ability compared with wild-type cells. Reduced *Epor* expression was detected on ∆EpoR_E_-osteogenic cells (Supplementary Fig. 3c), and they did not respond to EPO treatment (Fig. 4g, h). These data suggest a direct effect of EPO on osteogenic cells. These observations are also consistent with the in vivo observations of reduced trabecular bone seen in ∆EpoR_E_ mice (Fig. 2) and unchanged bone architecture seen with EPO treatment in these mice (Fig. 2d–h).

**Fig. 5:**
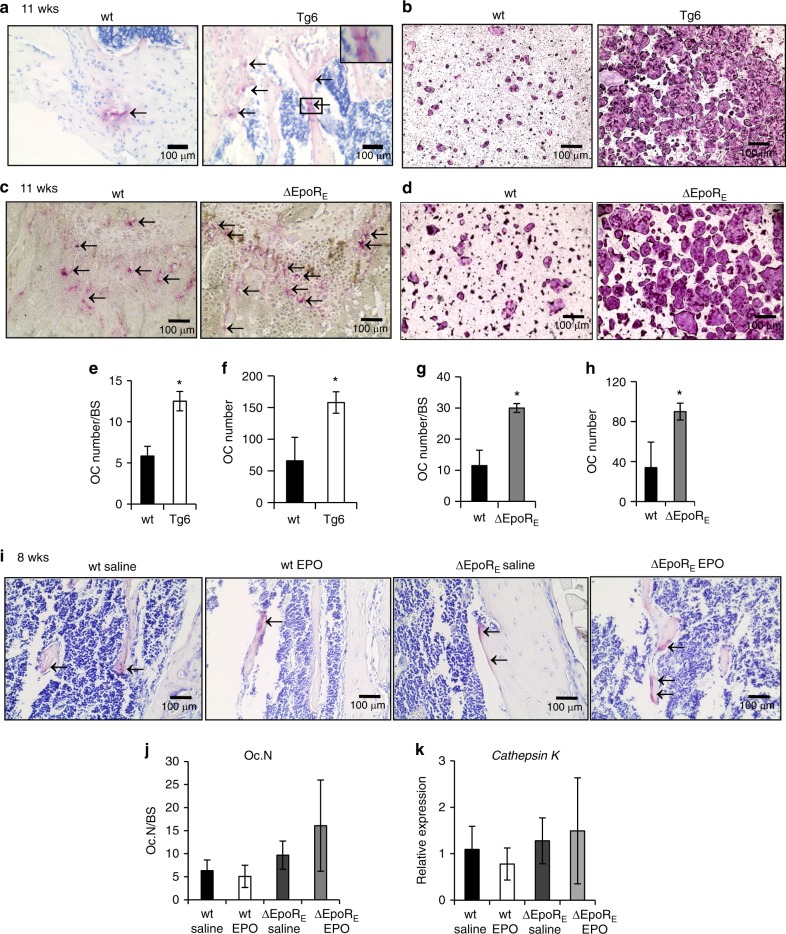
Osteoclasts in Tg6- and ΔEpoR_E_ mice. **a** Representative images of TRAP stained osteoclasts in the femur sections of 11-week-old wt and Tg6-mice. Arrows indicate purple stained osteoclasts along the surface of trabecular bones. **b** Images of in vitro osteoclast differentiation of bone marrow cells of wild-type (wt) and Tg6-mice using M-CSF and RANKL. Osteoclasts appear after 4–5 days and were stained for TRAP. **c** TRAP-stained sections of femurs of 11-week-old wild-type (wt) and ΔEpoR_E_-mice showing osteoclasts. Arrows indicate purple stained osteoclasts along the surface of trabecular bone. **d** In vitro osteoclast differentiation from the bone marrow of wt and ΔEpoR_E_-mice identified by TRAP staining. **e–h** Number of osteoclasts on the bone surface of wt and Tg6-mice (**e**), number of osteoclasts in the bone marrow cultures of wt and Tg6-mice (**f**), number of osteoclasts on the bone surface of wt and ΔEpoR_E_-mice (**g**), and number of osteoclasts in the bone marrow cultures of wt and ΔEpoR_E_-mice (**h**) analyzed using ImageJ. **i** TRAP stained femur sections of 8-week-old wt and ΔEpoR_E_-mice receiving 1200 U·kg^–1^ of EPO or saline for 10 days. Arrows indicate purple stained osteoclasts along the trabecular bone surface. **j** Enumeration of osteoclasts in the wt and ΔEpoR_E_-mice receiving EPO or saline. **k** Relative expression of *Cathepsin K* levels in the whole bone marrow determined by real-time PCR. (*n* = 5/group, *P < 0.05, **P < 0.01, ***P < 0.001)

Femurs of Tg6 mice analyzed by micro-Ct (Fig. 1) were stained for TRAP to detect osteoclasts and EPO overexpression in Tg6 mice was associated with increased osteoclasts (Fig. 5a, e). Bone marrow derived osteoclast cultures showed Tg6-cells formed numerous giant multinucleated osteoclasts (Fig. 5b, f). Tg6-osteoclasts expressed human EPO, but reduced *Epor* on day 4 of differentiation compared with wild-type osteoclasts (Supplementary Fig. 3d, e). Therefore, chronic EPO overexpression in Tg6 mice accelerates differentiation of osteoclasts and osteoblasts in vitro suggesting a high bone turnover rate in Tg6 mice.^19^

TRAP stained sections of femurs of 11-week-old ∆EpoR_E_-mice showed increased osteoclasts lining the bone surface (Fig. 5c, g). Consistent with in vivo observations, we found numerous multinucleated osteoclasts in bone marrow cultures of ∆EpoR_E_ mice (Fig. 5d, h). Osteoclasts of ∆EpoR_E_ mice expressed *Epor* comparable to wild-type osteoclasts (Supplementary Fig. 3f). Since, the *Epor* expression in ∆EpoR_E_ mice is *Gata1* dependent, we measured *Gata1* mRNA levels in FACS sorted preosteoclasts from the bone marrow and found both wild-type and ∆EpoR_E_-mice preosteoclasts expressing *Gata1* (Supplementary Fig. 3h). EPO treatment of bone marrow cultures from wild-type and ∆EpoR_E_-mice had no effect on their differentiation (Supplementary Fig. 3g). However, unlike 11-week-old ∆EpoR_E_ mice, young ∆EpoR_E_ mice of 8 weeks of age did not have any increase in osteoclasts in bone sections (Fig. 5i, j). Compared to age-matched wild-type mice, ∆EpoR_E_ mice gain increased fat mass and body weight with age and exhibit systemic inflammation^25^ as confirmed by significantly high C-reactive protein in the serum (Supplementary Fig. 1c). Therefore, the increase in osteoclasts seen in 11-week-old mice might be associated with high level of inflammation occurring in these mice with body weight gain. We did not observe any increase in osteoclasts in EPO treated wild-type mice (Fig. 5i, j). Whole bone marrow analysis showed no differences in *Cathepsin K* expression, a bone resorption marker secreted by osteoclasts (Fig. 5k).

### Elevated EPO inhibits bone morphogenetic protein 2 (BMP2)-induced bone formation

Since acute EPO treatment reduced bone without apparent changes in osteoclasts, we focused on the role of EPO in osteogenic differentiation. BMPs promote osteogenic differentiation; therefore, we assessed levels of several BMPs in whole bone marrow of wild-type, Tg6-, and ∆EpoR_E_-mice, and mice treated with EPO. Among the BMPs, we detected *Bmp2* and *Bmp6* in whole bone marrow and found Tg6 mice with chronic EPO had reduced *Bmp2* and *Bmp6* expression (Supplementary Fig. 4a, b). Elevated EPO also decreased *Bmp2* and *Bmp6* in bone marrow of wild-type mice, but not in ∆EpoR_E_ mice. However, saline treated ∆EpoR_E_ mice had low *Bmp2* levels in bone marrow (Supplementary Fig. 4c, d).

**Fig. 6:**
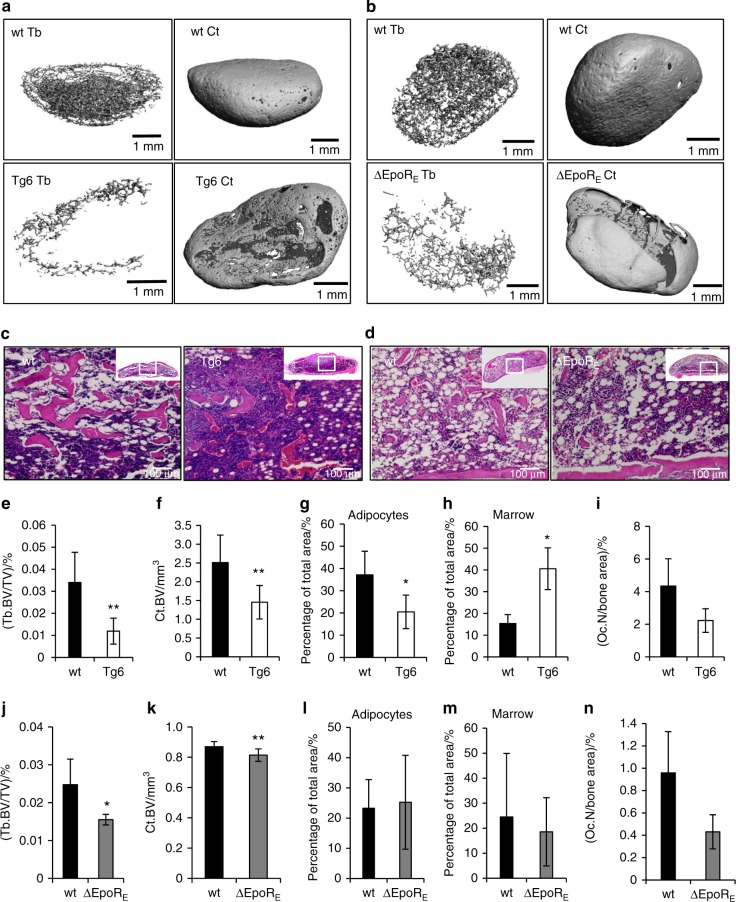
Elevated EPO and lack of EPOR signaling inhibit BMP2-induced ectopic bone formation. **a** Micro-CT images of ectopic bone formed by BMP2 in collagen scaffolds transplanted in wild-type (wt) and Tg6-mice 4 weeks post transplantation. Shown are ossicles formed in wt mice (top) and in Tg6-mice (bottom), and inner trabecular bone (right) and cortical bone (left). **b** Micro-CT images of ectopic bone formed by BMP2 in collagen scaffolds transplanted in wild-type (wt) and ΔEpoR_E_-mice 4 weeks post transplantation. Shown are ossicles formed in wt mice (top) and in ΔEpoR_E_-mice (bottom), and inner trabecular bone (right) and cortical bone (left). **c** H&E staining of decalcified ossicles formed in the wt and Tg6-mice, showing bone as pink colored regions, adipocytes as white circular regions and marrow as blue regions. **d** H&E stained images of ossicles formed in wt and ΔEpoR_E_-mice bone, marrow and adipocytes. **e**, **f** Micro-CT quantification of trabecular (**e**) and cortical (**f**) volume of the ectopic bone formed in wt and Tg6-mice. **g** Quantification of marrow adipocytes in the wt and Tg6-ossicle sections. **h** Space occupied by marrow (excluding adipocytes and bones). **i** Number of TRAP stained osteoclasts in the wt and Tg6-ossicles. **j**, **k** Micro-CT quantification of trabecular (**j**) and cortical (**k**) volume of the BMP2-induced ectopic bone formed in wt and ΔEpoR_E_-mice. **l** Quantification of marrow adipocytes in the wt and ΔEpoR_E_-ossicle sections. **m** Space occupied by marrow (excluding adipocytes and bone). **n** Number of TRAP stained osteoclasts in the wt and ΔEpoR_E_-ossicles. Quantification of adipocytes, marrow space and osteoclast numbers were performed using ImageJ analysis. (*n* = 4–5/group, *P < 0.05, **P < 0.01)

BMP2 is a potent osteogenic factor^26^ and has also been shown to promote adipogenesis.^27,28^ To probe EPO–BMP2 interaction in bone development, a BMP2-induced ectopic bone formation assay was performed by subcutaneous implantation of collagen sponges soaked with 5 μg of BMP2 into Tg6 mice, wild-type littermates, ∆EpoR_E_ mice, and wild-type controls. Local inducible precursors expressing BMP receptors interact with the BMP2 in the sponges and inducible precursors in skin will develop into bone. Thus, the collagen scaffold with BMP2 will result in formation of a bone/marrow organ with outer cortical and inner trabecular bone interspersed with marrow cells and adipocytes. At 4 weeks post transplantation, BMP2-induced-ossicles were harvested for micro-CT scans and histology analysis. Ossicles formed in Tg6 mice (Fig. 6a) had reduced trabecular and cortical BV (Fig. 6e, f) compared to those formed in littermate control. Histology analysis (Fig. 6c) revealed extensive erythropoiesis, less bone, fewer adipocytes (Fig. 6g), and increased marrow (Fig. 6h) in Tg6-ossicles. Features in ossicles formed in Tg6 mice were consistent with reduced bone and marrow adipocytes, and hypercellular marrow observed in these mice (Figs. 1 and 3a). Analysis of TRAP stained sections of ossicles showed fewer osteoclasts in Tg6-ossicles (Fig. 6i), suggesting that reduced bone in Tg6-ossicles is not due to resorption by osteoclasts. This is in contrast to the increased osteoclast number and bone reduction observed in Tg6 femur sections (Fig. 5a, e). Ossicles formed in ∆EpoR_E_ mice (Fig. 6b) also had lower trabecular and cortical BV (Fig. 6j, k) compared with ossicles that developed in control mice. These features were consistent with the reduced bone phenotype observed in ∆EpoR_E_ mice (Fig. 2). Histology analysis (Fig. 6d) of ∆EpoR_E_-ossicles did not show any significant differences in adipocytes, marrow, and in the number of osteoclasts found on bone surface (Fig. 6l–n).

### Elevated EPO signaling in BMSCs reduces their differentiation into osteoblasts and adipocytes

We performed colony formation efficiency assays with bone marrow cultures of wild-type, Tg6-, and ∆EpoR_E_-mice (*n* = 5–6/group) to assess the colony forming potential of BMSCs present in the bone marrow. BMSCs from Tg6 mice showed reduced colony forming ability (Supplementary Fig. 5a), whereas we did not observe any defect in the colony forming efficiency in the BMSCs from the ∆EpoR_E_ mice (Supplementary Fig. 5b). Since in vitro culture conditions lack the extensive regulatory systems present in vivo, to further investigate EPO regulation of BMSC differentiation into osteoblasts or adipocytes we performed in vivo ectopic bone formation studies following BMSC transplantation into immunodeficient NSG mice.

**Fig. 7:**
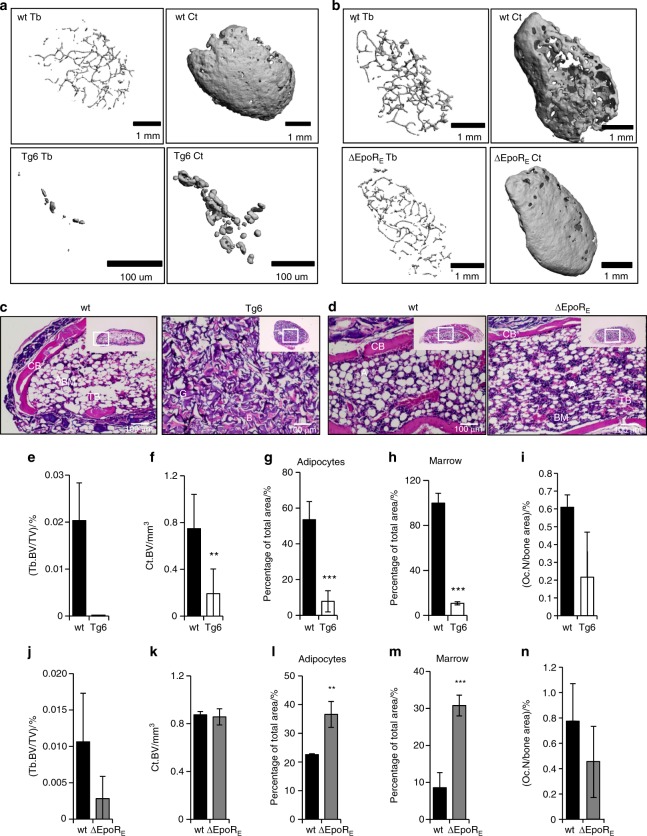
In vivo bone formation potential of BMSCs from Tg6- and ΔEpoR_E_-mice transplanted in immunocompromised mice. **a** Micro-CT images of ectopic bone formed by the differentiation of BMSCs from wild-type (wt) and Tg6-mice 4 weeks post transplantation. Shown are wt BMSC ossicles (top) and Tg6-BMSC ossicles (bottom), and inner trabecular bone (right) and cortical bone (left). **b** Micro-CT images of ectopic bone formed by BMSCs from wild-type (wt) and ΔEpoR_E_-mice. Shown are wt BMSC ossicles (top) and ΔEpoR_E_-BMSC ossicles (bottom), and inner trabecular bone (right) and cortical bone (left). **c**, **d** H&E staining of wt, Tg6 (**c**), and ΔEpoR_E_ (**d**) BMSC ossicles showing cortical bone (CB), trabecular bone (TB), adipocytes (A), gelfoam (G), and bone marrow (BM). **e**, **f** Quantification of trabecular (**e**) and cortical bone volume (**f**) of wt and Tg6-BMSC ossicles. **g**, **h** Area of adipocytes (**g**) and bone marrow (**h**) in wt and Tg6–BMSC ossicles quantified using ImageJ software analysis. **i** Quantification of the number of osteoclasts present in the ossicles developed from the wt and Tg6-BMSCs. **j**, **k** Micro-CT quantification of trabecular (j) and cortical bone volume (**k**) of ossicles developed from the differentiation of wt and ΔEpoR_E_-BMSCs. **l**, **m** Area of adipocytes (**l**) and bone marrow (**m**) inside the wt and ΔEpoR_E_-ossicles. **n** Quantification of osteoclasts present on the wt and ΔEpoR_E_-BMSC ossicles. (*n* = 4/group, **P < 0.01, ***P < 0.001)

Isolation of BMSCs by culturing adherent cells of bone marrow followed by depletion of CD45^+^ CD11b^+^ cells by magnetic sorting to exclude all hematopoietic cells including contaminating macrophages has been shown to yield pure BMSCs that can be used for in vivo bone formation assays.^29^ These cells express positive BMSC markers, which are stable for cell passages until 8–10. We used these validated protocols to isolate BMSCs from wild-type, Tg6-, and ∆EpoR_E_-mice (*n* = 4–5/group), to perform in vivo bone formation assays.^30^ BMSCs from Tg6 mice express human EPO, and also express *Epor* in comparable levels to that of wild-type littermates (Supplementary Fig. 4e, f). ∆EpoR_E_–BMSCs do not express *Epor* (Supplementary Fig. 4g). Isolated BMSCs were absorbed into a collagen scaffold and surgically transplanted subcutaneously into immunodeficient mice. Each mouse received one each of wild-type and Tg6- or ∆EpoR_E_-mice transplants, as well as one collagen sponge without any cells. In this assay, BMSCs will differentiate and form a bone/marrow organ (Fig. 7a–d) consisting of bone, adipocytes, and stroma of donor origin, whereas the hematopoiesis originates from the recipient mice.^31^ BMSCs from each mouse were transplanted into a minimum of three different recipient mice to obtain consistent results. Micro-CT analysis of ossicles after 8 weeks post transplantation showed reduced osteogenic potential of Tg6–BMSCs compared to wild-type (Fig. 7a). Histology analysis showed that Tg6–BMSCs ossicles did not develop into well-defined cortical and trabecular bone structures as seen in ossicles formed from wild-type BMSCs (Fig. 7c). There were hardly any trabecular features in most of the ossicles formed by Tg6–BMSCs and, for this reason, trabecular parameters could not be calculated for Tg6–BMSCs (Fig. 7e). Analysis of cortical bone formation showed Tg6-ossicles having less cortical BV (Fig. 7f). Tg6–BMSCs also had reduced adipogenesis and marrow formation (Fig. 7c, g, h). There was no change in hematocrit levels of recipient mice transplanted with Tg6–BMSCs (data not shown). There were also no significant differences in number of osteoclasts in Tg6-ossicles (Fig. 7i). Ossicles formed by ∆EpoR_E_–BMSCs had a similar structure to that of control (Fig. 7b, d); however, separation of cortical and trabecular bone suggested ossicles formed by ∆EpoR_E_–BMSCs had a trend for fewer trabeculae (Fig. 7j) with no change in cortical BV (Fig. 7). ∆EpoR_E_ mice had enhanced adipogenic differentiation capacity and enhanced marrow (Fig. 7l, m). Analysis of osteoclasts showed no differences in ossicles formed by ∆EpoR_E_–BMSCs when compared with wild-type controls (Fig. 7n).

**Fig. 8:**
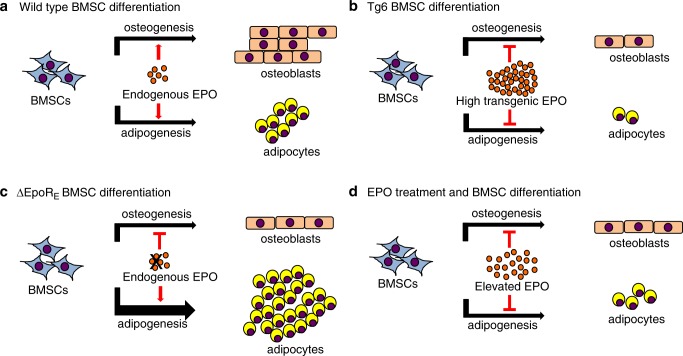
Schematic for erythropoietin regulation of BMSC differentiation. **a** EPO-EPOR signaling in BMSCs is required for maintaining the balance between osteogenic and adipogenic differentiation in the marrow. **b** Elevated EPO signaling in Tg6-mice inhibits both osteogenesis and adipogenesis. **c** Absence of endogenous EPO signaling in BMSCs in ΔEpoR_E_-mice reduces osteogenesis and favors adipogenesis resulting in fatty marrow. **d** EPO treatment in mice inhibits both osteogenesis and adipogenesis

Gene expression analysis of Tg6–BMSCs used for ectopic bone formation assay showed reduced *Ppar-*γ and *Osterix* expression, which is consistent with reduction in both adipogenesis and osteogenesis (Suppl Fig. 4h). Tg6–BMSCs also expressed more *Gata2* and *Gata3*. Increased *Gata2* and *Gata3* have been reported to inhibit *Ppar-*γ and suppress adipogenesis.^32^ ∆EpoR_E_–BMSCs had more *Ppar-*γ expression and reduced *Gata3* levels (Supplementary Fig. 4i), which is consistent with their increased adipogenesis. Unlike Tg6–BMSCs with reduced *Osterix* expression, in ∆EpoR_E_–BMSCs *Osterix* expression was elevated and ossicle formation was similar to wild-type BMSC. We assessed the expression of *Epor* in BMSCs relative to other cells and tissues that express *Epor*. Even though, the *Epor* levels are low in BMSCs, our data show intrinsic EPO signaling as an essential factor for their differentiation (Supplementary Fig. 5c). Therefore, endogenous EPO is required to maintain BMSC balance between osteogenesis and adipogenesis while high EPO inhibits both osteogenic and adipogenic differentiation (Fig. 8).

## Discussion

In this study, we addressed the role of EPO signaling in bone, BMSCs and bone marrow adipocytes in Tg6 mice with chronic elevated EPO, wild-type mice with acute EPO treatment, and ∆EpoR_E_ mice lacking EPO signaling in non-erythroid cells. We recently observed a sex-differential role of EPO in metabolic regulation where elevated EPO reduced external fat depots in male mice but not in female mice.^33^ Here, we find that reduction in marrow fat with elevated EPO is not gender-specific as both male and female Tg6-mice had similar reduction in marrow fat. However, we noted gender differences might contribute to some of the variations in trabecular parameters we observed in male Tg6 mice compared with previous report for female Tg6 mice.^22^ In our study, male Tg6 mice had extensive reduction of trabecular bone with decreased trabecular thickness, whereas Tg6 female mice had increased trabecular thickness. Only male Tg6-mice had reduced femoral length and body weight, whereas female Tg6-mice did not differ from their littermates in these parameters.

We report that endogenous EPO is important for bone development and marrow adipogenesis as ∆EpoR_E_ mice had reduced trabecular bone and increased marrow adipocytes, and these features amplified with age. In mice, age related changes in bone microarchitecture occur early in life with peak trabecular BV between the ages of 6 and 8 weeks followed by a steady decline specifically in the long bone metaphyses.^34^ In our study, in the younger ∆EpoR_E_ mice of 8 weeks of age, the absence of endogenous EPO signaling showed early signs of reduction in trabecular bone with fewer trabeculae and increased spacing. However, at 11 weeks of age, reduction in trabecular bone becomes apparent with significant reduction in trabecular BMD and trabecular BV. The ΔEpoR_E_-mice gains body weight and fat mass with age because of lack of Epo signaling in non-hematopoietic tissues.^8^ Increased fat mass is associated with lower BMD in mice^35^ and in humans obesity is reported to show low bone formation.^36^ Compared to body weight matched wild-type mice, the ΔEpoR_E_-mice had reduced trabecular bone ruling out the role of body weight as a factor for reduced bone phenotype. But, we cannot exclude the possibility of increased fat mass in the 11-week-old ΔEpoR_E_ mice contributing to a low bone phenotype in our study.

Endogenous EPO is also important in osteoclast differentiation particularly as mice become older as we observed increased osteoclasts in the 11-week-old ∆EpoR_E_ mice. These mice also have systemic inflammation^25^ and gain body weight with age. Therefore, the increase in osteoclasts seen in 11-week-old mice might be associated with their high level of inflammation consistent with previous reports linking increased osteoclast differentiation to inflammation.^37^ In mice models, induction of systemic inflammation by methods like high-fat diet feeding^35^ or administration of lipopolysaccharide is associated with enhanced osteoclastogenesis causing bone resorption resulting in bone loss.^38^ Thus, the reduction in bone seen in the 11-week-old ∆EpoR_E_ mice could also be a consequence of inflammation arising due to the lack of Epo signaling in non-hematopoietic tissues. Therefore, we assessed bone parameters in younger ∆EpoR_E_ mice 8 weeks of age where their body weight is similar to control mice. In young ∆EpoR_E_ mice we still observed reduction in trabecular bone, but there was no increase in osteoclasts or increased *Cathepsin K* suggesting the initial disruption of trabecular bone occurring in young ∆EpoR_E_ mice as an osteoclast-independent mechanism. Thus our data show endogenous EPO as an essential cytokine with varying levels of regulation in the bone environment. In younger mice with rapid bone development as well as in older mice with age induced skeletal disruption endogenous EPO is required for normal bone development particularly in the maintenance of trabecular bone microarchitecture.

Exogenous EPO administration increases hematocrit in a dose dependent manner^39^ and in mice both low dose (300 U·kg^−1^)^19^ and high dose (9 000 U·kg^–1^)^22^ of EPO administration reduced trabecular bone in long bones. However, whether EPO-induced bone loss is associated with EPO stimulated erythropoiesis or is entirely mediated by non-erythroid cells is not known. Therefore, we injected young wild-type and ∆EpoR_E_ mice 6 weeks of age with 1 200 U·kg^–1^ rhEPO for 10 days to achieve increased hematocrit during the period of maximum bone remodeling in mice. Unlike wild-type mice, EPO treatment in ∆EpoR_E_ mice did not result in trabecular bone loss despite increasing hematocrit levels comparable to EPO treated wild-type mice. This provides evidence that reduced bone parameters seen with elevated EPO is independent of erythropoiesis and is mediated by non-erythroid cell response.

The role of endogenous EPO signaling in marrow adipogenesis has not been reported. The increased marrow adiposity in ∆EpoR_E_ mice shows that lack of EPO signaling in non-erythroid cells leads to a fatty marrow. The ∆EpoR_E_ mice gain increased body weight because of accumulation of white fat mass and this accrual increases with age.^8^ In younger ∆EpoR_E_ mice of 8 weeks of age, we observed significant increase in marrow adipocytes even before these mice started to gain weight compared to the age-matched wild-type mice. These data show endogenous EPO signaling as an important regulator of marrow adiposity. In the bone marrow of female mice there was extensive adiposity compared with age-matched male mice. A steep increase in marrow adiposity is also observed in menopausal women with 10% more vertebral marrow fat than men of similar age.^40^ The reduction in marrow adipocytes in both wild-type and ∆EpoR_E_-mice with EPO administration shows that this process is due to EPO stimulated erythropoiesis in the marrow. Thus, EPO regulation of bone marrow adipocytes is different from EPO regulation of adipocytes in external fat depots. As previously reported, ∆EpoR_E_ mice that do not express *Epor* in adipose tissue do not lose white fat mass with EPO treatment despite increased hematocrit.^8^ Loss of bone marrow adipocytes with EPO treatment does not depend on the presence of *Epor* on these cells as both wild-type and ∆EpoR_E_-mice lose bone marrow adipocytes. However, endogenous EPO–EPOR signaling is important for regulated bone marrow adipogenesis because its absence results in fatty marrow in ∆EpoR_E_ mice. These data suggest that the reduction in bone and marrow fat by elevated EPO is mediated differently. With bone loss, we observe an erythropoietic independent action of EPO, whereas reduction in marrow adipocytes is associated with EPO stimulated increased erythroid cell production in the marrow.

The reduced bone observed in Tg6 mice and in wild-type mice receiving EPO has been associated with increased osteoclasts.^22^ Our findings in Tg6-mice were similar to the previous report, but we did not observe osteoclast induction in mice with acute EPO treatment despite bone loss. Bone loss in wild-type mice treated with EPO without an increase in osteoclasts was also previously observed.^41^ Similarly, addition of EPO did not stimulate osteoclast differentiation in vitro, consistent with report on lack of EPO effect in in vitro osteoclast cultures.^19^ Thus, elevated EPO in chronic and acute conditions reduce trabecular bone possibly by different mechanisms. Increase in osteoclasts with chronic elevated EPO can contribute to bone reduction, but is less evident in acute EPO treatment. The response of osteoclasts to chronic EPO is known and we also observed increased osteoclasts in the Tg6-mice bones and marrow cultures, but the osteoblast response to chronic EPO in the Tg6-mice has not been previously reported. We observed increased *Alp*, *Osterix* expression in the primary osteogenic cells from these mice along with increased mineralization in cultures. Early reports of analysis of bone markers in Tg6 mice identified increased serum TRAP and reduced serum Osteocalcin levels,^22^ suggesting a high bone resorption and low bone formation rate. Based on gene expression and in vitro osteogenic cultures from our studies along with information on the bone markers in Tg6 mice, it can be concluded that chronic exposure to high levels of EPO results in both increased osteoclast and osteoblast activity causing high bone turnover resulting in bone reduction.

We find elevated EPO reducing *Bmp* levels in both chronic and acute conditions. *Bmp2* and *Bmp6* are important osteogenic factors,^42^ produced in the bone marrow by macrophages, osteoblasts, and chondrocytes.^43–45^ Expression of EPOR by all these cells^20,46^ raises the potential for regulation of BMP signaling by EPO. In Tg6 mice, high EPO levels inhibited the ectopic bone formation potential of BMP2, in an osteoclast independent manner. In this assay, ossicles formed in Tg6 mice were exposed to elevated EPO only for 4 weeks, a duration similar to acute EPO treatment in mice. In both these conditions, increased EPO did not increase osteoclasts but reduced bone suggesting that the primary response of elevated EPO in bone is not to stimulate bone resorption but to inhibit bone formation. Elevated EPO exposure for a prolonged period of time might eventually increase osteoclasts and contribute to reduced bone as seen in Tg6 mice. Previous studies showed EPO synergistically interacting with BMP2 to promote bone formation^47,48^ in calvarial defect models where EPO promotes tissue repair by augmenting BMP2 induced osteogenesis. In addition to reducing bone formation, we observed that elevated EPO increased marrow and reduced adipocytes in ectopic bone, consistent with an earlier report.^48^ In the ∆EpoR_E_ mice, we observed reduced *Bmp2* in the marrow and a reduction in BMP2-induced ectopic bone formation. Therefore, both elevated EPO and disrupted endogenous EPO signaling inhibits BMP2-induced osteogenesis and aberrant BMP signaling could be one mechanism by which osteogenesis is impaired under these conditions.

Precursors of osteoblasts in the postnatal organism are BMSCs, and we examined the function of EPO signaling on BMSC differentiation in conditions of elevated EPO and in the absence of endogenous EPO signaling using in vivo bone formation assays. Tg6-BMSCs with increased EPO signaling had limited differentiation ability for both osteoblast and adipocyte lineages. These cells have increased *Gata2* and *Gata3* expression in the Tg6-BMSCs with reduced *Ppar-*γ expression. GATA2 and GATA3 have been shown to inhibit PPAR-γ activity and thereby reduce adipogenesis.^49^ The essential role of endogenous EPO signaling in BMSC differentiation is evident from the wild-type BMSCs expressing *Epor*, which formed ectopic bone with distinct trabecular and cortical bone with well distributed adipocytes in the marrow space. Conversely, BMSCs from ∆EpoR_E_ mice, which do not express *Epor*, and therefore do not respond to circulating EPO in the recipient mouse, formed bone with less trabeculae and more adipocytes. Report of osteoblast production of EPO directly modulates erythropoiesis raising the possibility of EPO/EPOR autocrine response even with low *Epor* expression in osteoblasts.^50^ Furthermore, a direct effect of EPO in cultures of primary mouse BMSCs was indicated by treatment with low dose EPO that inhibited expression of osteoblast specific genes, *Alp*, *Runx2*, *and Osteocalcin* while siRNA silencing of *Epor* completely abrogated the reduction of osteoblast markers.^41^ In cultures of human BMSCs, EPO treatment increased cell viability, ALP activity, and cell mineralization dependent on JAK2, PI3K, and mTOR pathways, which was inhibited by mTOR inhibitor Rapamycin.^51^ Similarly, Rampamycin also was reported to inhibit EPO induced osteogenic differentiation of human BMSC cultures.^52^

Increased *Ppar-*γ expression in ∆EpoR_E_-BMSCs is consistent with their increased adipogenic differentiation potential. In vitro differentiation studies using BMSCs have shown EPO promoting their differentiation to osteoblasts.^52^ We performed in vitro differentiation assays with whole bone marrow cultures, but had frequent macrophage overgrowth during the long-term culture conditions required for differentiation assays. Differentiation assays with sorted primary BMSCs also were not possible as the BMSCs did not survive by themselves after couple of days in culture. Our data show that the EPO effect in BMSC differentiation in vivo is different from in vitro culture conditions reported previously. The ectopic bone/marrow formation assay with BMSCs also reflects the bone architecture of the mouse models we used; Tg6–BMSCs have decreased osteogenesis and adipogenesis and Tg6 mice have reduced bone and marrow adipocytes (Figs. 1 and 3). ∆EpoR_E_–BMSCs developed less trabecular bone and more bone marrow adipocytes consistent with the bone in these mice (Figs. 2 and 3). Thus, absence of EPO signaling disproportionately increases the differentiation of BMSCs to marrow adipocytes with reduction in osteogenesis, while high EPO signaling in BMSCs decreases their differentiation to either lineages.

In conclusion, we provide evidence for the essential role for both endogenous EPO and elevated EPO signaling in the development of bone and marrow adipocytes. Disrupted differentiation of BMSCs due to altered cytokine signals in the marrow environment enhances adipogenesis while diminishing the osteogenic potential of BMSCs.^53–55^ Bone marrow adipocytes have a distinct origin and function compared with adipocytes comprising white and brown adipose tissue.^56,57^ With recent improvements in the measurement of bone marrow adipocytes in humans^58^ and in animal models,^59^ studies addressing the role of EPO signaling in the bone marrow fat compartment will shed new insights in understanding the non-hematopoietic roles of EPO in development and disease.

## Methods

### Mice models

Tg6 mice overexpressing PDGFβ promoter driven human EPO^23^ and ΔEpoR_E_ mice that are *Epor*-/- mice rescued by erythroid restricted *Epor* transgene (GATA-1 locus hematopoietic regulatory domain driving mouse *Epor* cDNA),^24^ established on C57BL6/J background, were used as models for chronically elevated EPO and lack of EPOR signaling in non-erythroid cells, respectively. Wild-type mice (C57BL6/J) were obtained from (Jackson Laboratory, ME, USA). Recombinant human epoetin-α (Amgen, CA, USA) was administered to wild-type and ΔEpoR_E_-mice subcutaneously at a dose of 1 200 IU·kg^–1^ daily for 10 days. Control mice were injected with phosphate buffered saline (PBS) (Gibco, Life Technologies, USA). For SSC/BMSC transplantation, 8-week-old female immunocompromised NSG mice (NOD.Cg-PrkdcscidIl2rgtm1WjI/SzJ; Jackson Laboratory, ME, USA) were used. All animal procedures were conducted under the National Institutes of Health guidelines.

### Micro-CT analysis

Mouse femurs were analyzed using a Bruker Skyscan 1172 micro-CT scanner (Micro Photonics Inc, PA, USA). Trabecular and cortical regions were analyzed at 0.35 mm and 4.25 mm from the growth plate, respectively. For both regions, 1.5 mm of femur bone sections comprising 257 bone slices were individually analyzed using CTAn software at 6 μm resolution and 3D models of regions of interest were constructed. The regions of interest were similar in 8-week and 11-week-old mice analyzed in this study.

### Bone histology

Mouse femurs were fixed in 10% buffered neutral formalin (Sigma, MO, USA) for 24 h followed by decalcification in 10% EDTA (0.5 mol·L^–1^) at 4 °C until complete decalcification was confirmed by x-ray analysis. Hematoxylin–Eosin (H&E) staining was performed on 6 μm sections of decalcified paraffin embedded femurs to assess bone marrow histology. For osteoclast detection in femurs, Tartrate-resistant acid phosphatase (TRAP) staining was performed using TRAP/ALP Stain Kit according to the manufacturer’s instructions (#294-67001, Wako Chemicals, USA).

### Osteoclast generation from bone marrow

Bone marrow cells were harvested from 8- to 10-week-old mice and seeded into tissue culture treated plates and allowed to attach overnight in α-MEM medium with 10% FBS. Nonadherent cells (5 × 10^5^ cells/cm^2^) were plated into 96 flat-bottomed well plates in fresh medium containing 30 ng·mL^–1^ RANKL and 20 ng·mL^–1^ M-CSF (Peprotech, NJ, USA). Medium was replaced every other day until osteoclasts appeared (4–5 days). TRAP staining was performed according to manufacturer’s instructions (#387-1A, Sigma, St Louis, MO, USA) to visualize osteoclasts.

### Osteogenic cultures from calvaria

Calvarial osteogenic cells were isolated from 6 to 8 weeks old mice using previously described protocol.^60^ Briefly, calvarial pieces without red hematopoietic regions were placed in solution of collagenase II and dispase (4 mg·mL^–1^ each) in PBS (Worthington Biochemical Corp, NJ, USA). Calvarial pieces were incubated for 5 × 15 min changes total of collagenase II and dispase solution on a shaker at 37 °C. Cells obtained from the first 15 min incubation were discarded to exclude hematopoietic cells. Cells from the remaining four changes were pooled in tubes of fresh α-MEM medium with 20% FBS, centrifuged and plated in α-MEM medium with 20% FBS in 25 cm^2^ tissue culture flask. Fresh medium was replaced the next day followed by medium change twice weekly. Once cells reach confluence (1–2 weeks), they were incubated in medium containing 5 mmol·L^–1^ β-glycerophosphate and 100 μg·mL^–1^ ascorbic acid (Sigma, MO, USA) with medium replacement twice weekly.

### Viability, differentiation and mineralization assays

Viability of osteogenic cells was measured using Thiazolyl Blue Tetrazolium Blue (MTT) assay (Sigma). Alkaline phosphatase (ALP) activity was quantified using SIGMAFAST™ p-Nitrophenyl phosphate Tablets (Sigma). For microscopic observation of ALP activity, SIGMA FAST™ BCIP/NBT (5-Bromo-4-chloro-3-indolyl phosphate/Nitro blue tetrazolium) tablets were used according to manufacturer’s instructions. Mineralization was detected using 1% alizarin staining (pH 4.6). All reagents for measuring ALP activity, ALP visualization and mineralization were purchased from Sigma, MO, USA.

### Colony forming unit assay

Estimation of SSCs in the BMSC population was determined by colony forming efficiency assay as described previously.^29^ Briefly, 5 × 10^5^ nucleated cells from bone marrow following red blood cell lysis, were plated into 25 cm^2^ culture flask in triplicates containing 6 mL α-MEM medium with lot-selected non-heat-inactivated 20% FBS and 0.1 mmol·L^–1^ of β-mercaptoethanol. Cells were incubated for 14 days at 37 °C in a humidified atmosphere with 5% CO_2_. Cells were washed with PBS, fixed with methanol and stained with 0.5% crystal violet. Only colonies consisting 50 or more cells are used for determining colony forming efficiency.

### FACS

For isolating preosteoclasts to measure *Gata1* mRNA levels, bone marrow cells after red blood cell lysis were incubated with 0.5 μg of anti-mouse CD16/CD32 antibody on ice for 5 min for blocking Fc receptors. Cells were stained for the following mouse antibodies; mouse CD45-APC, CD14-FITC, CD11b-Alexa-780, and M-CSF receptor-PE conjugated antibodies (ThermoFisher; Waltham, MA, USA) according to the manufacturer’s directions. Cells were sorted using FACS calibur flow cytometer (BD Bioscience) and osteoclasts were identified as CD45^+^/CD14^+^/CD11b^+^/MSCFR^+^ cells.

### Real-time PCR

Total RNA was extracted using RNeasy Mini kit (Qiagen, MD, USA) and treated with DNase I (Promega, WI, USA); 1–2 μg was reverse transcribed using MultiScribe Reverse Transcriptase (ABI) (Thermo Fisher Scientific, MA, USA) for quantitative PCR assays. For mouse *Epor* analysis, Taqman primers and probe and mouse *S16* primers and probe (house-keeping gene) were used as described previously.^8^ For all other PCR assays, SYBR green real-time PCR was carried out using gene specific primers (Supplementary Table 1) with normalization to house-keeping gene β*-Actin* using SYBR Green dye and qPCR SuperMix (Roche Applied Science, IN, USA); relative mRNA quantification was calculated by delta-delta Ct method.

### Ectopic bone formation assays with BMP2

Eight-week-old Tg6 mice and littermate controls, ΔEpoR_E_- and wild-type mice were transplanted subcutaneously with 5 μg of BMP2 (#34-8507-85, Ebioscience, CA, USA) soaked into gelatin sponge cubes (7 × 5 × 5 mm) (Gelfoam™, Pfizer, NY, USA). After 4 weeks ectopic bone formation was confirmed using x-ray analysis (In vivo Xtreme, Bruker) and transplants were harvested. After 24 h fixation in 10% neutral buffered formalin, transplants were moved to PBS and analyzed by micro-CT. Following decalcification in 0.05 mol·L^–1^ EDTA, H&E staining, and TRAP staining procedures were performed.

### Ectopic bone formation assays with BMSCs

Bone marrow harvested from 8-week old Tg6- and littermate controls, ΔEpoR_E_-, and wild-type mice, was made into a single cell suspension and plated in 6 mL α-MEM medium with lot-selected nonheat inactivated 20% FBS in a 25 cm^2^ flask and incubated undisturbed for 7 days. The adherent cell layer was washed with PBS, treated with 1 mg·mL^–1^ filter sterilized collagenase II solution for 15 min. The monolayer was washed again with PBS and incubated with 1% trypsin for 5 min. Cells were dislodged using a sterile cell scraper and plated in a 75 cm^2^ flask with α-MEM medium with lot-selected nonheat inactivated 20% FBS and incubated until confluent. Cells were passed into a 175 cm^2^ flask with α-MEM medium with lot-selected nonheat inactivated 20% FBS and grown until confluent. SSCs/BMSCs were separated from contaminating hematopoietic cells by negative selection using CD45 and CD11b magnetic beads (Miltenyi Biotec, CA, USA). For each transplant, 2 million BMSCs were suspended in 20 µL culture medium and absorbed into 7 × 5 × 5 mm gelatin sponge cubes (Gelfoam™, Pfizer, NY, USA) and implanted subcutaneously in the back of 8-week-old female immunocompromised NSG mice (NOD.Cg-PrkdcscidIl2rgtm1WjI/SzJ; Jackson Laboratory, ME, USA) to form ectopic bone with marrow. For determining hematocrits, blood was collected from tail vein of mice using heparin coated capillary tubes, centrifuged in a micro-hematocrit centrifuge (Unico, Dayton, NJ, USA). Hematocrit was measured using a VIN micro-hematocrit capillary tube reader (Veterinary Information Network Bookstore, Davis, CA, USA). After 8 weeks post transplantation, ectopic bone formation was confirmed by x-ray monitoring and transplants were harvested, fixed in 10% neutral buffered formalin. Transplants were moved to PBS and analyzed by micro-CT. For histology, fixed transplants were decalcified in 0.05 mol·L^–1^ EDTA in PBS for 2–3 weeks, paraffin embedded, and sectioned. H&E staining and TRAP staining were performed on sections to assess histology and to detect osteoclasts, respectively. Images were analyzed using ImageJ software to determine adipocyte and marrow area relative to total tissue area.

### Serum analysis and hematocrit measurement

Blood was collected terminally by cardiac puncture and levels of mouse EPO and human EPO in serum were measured using Quantikine ELISA assays (mouse EPO #MEP00B; human EPO #DEP00, R&D Biosystems, MN, USA) according to manufacturer’s instructions. Mouse C-reactive protein in serum was measured using ELISA assay (#ab157712, Abcam, Cambridge MA, USA). For hematocrit, blood was collected from the tail vein in heparin coated capillary tubes. The tubes were centrifuged using a micro-hematocrit centrifuge (Unico, NJ, USA) and hematocrits were measured using a VIN micro-hematocrit capillary tube reader (Veterinary Information Network Bookstore, CA, USA).

### Statistical analysis

Data are expressed as mean ± standard deviation. Two-tailed nonpaired Student’s *t* test was used to determine statistical significance between control and test groups. Comparisons among multiple groups were evaluated using one way analysis of variance with Dunnet’s multiple comparison post hoc tests at α = 0.05 (Graphpad Prism 6).

## Supplementary information

Supplementary Material Revised Marked

Supplementary Table 1

Supplementary Figure 1

Supplementary Figure 2

Supplementary Figure 3

Supplementary Figure 4

Supplementary Figure 5
