# Supplementary material for: Erythropoietin modulates bone marrow stromal cell differentiation
Source: Bone Res. 2019 Jul 25;7:21. doi: 10.1038/s41413-019-0060-0 (PMC6804931; doi:10.1038/s41413-019-0060-0)
Supplement: Supplementary file 1 — Supplementary Material Revised Marked [file 41413_2019_60_MOESM1_ESM.docx]

**Supplemental table 1. Primer and probe sequences**

| **Primers and probes** |  | **Sequences** |
| --- | --- | --- |
| *Alkaline phosphatase* | FP | TTGTGCCAGAGAAAGAGAGAGA |
|  | RP | GTTTCAGGGCATTTTTCAAGGT |
| *Runx2* | FP | ACGAAAAATTAACGCCAGTCG |
|  | RP | TCGGTCTGACGACGCTAAAG |
| *OsteriX* | FP | GAGGAAGAAGCTCACTATGGCTCCAG |
|  | RP | GCCTCCTTTCCCCAGGGTTGTTGA |
| *Bmp2* | FP | CACACAGGGACACACCAACC |
|  | RP | CAAAGACCTGCTAATCCTCAC |
| *Bmp6* | FP | AGCACAGAGACTCTGACCTATTTTTG |
|  | RP | CCACAGATTGCTAGTTGCTGTGA |
| *Cathepsin K* | FP | TAGCCACGCTTCCTATCCGA |
|  | RP | TCCTCCGGAGACAGAGCAAA |
| *β-actin* | FP | GCAGGAGTACGATGAGTCCG |
|  | RP | ACGCAGCTCAGTAACAGTCC |
| *human EPO* | FP | GGAGGCCGAGAATATCACGAC |
|  | RP | CCCTGCCAGACTTCTACGG |
| *Cebp-α* | FP | GCGGGAACGCAACAACATC |
|  | RP | GTCACTGGTCAACTCCAGCAC |
| *Cebp-β* | FP | GCAGCCACTTGAGTTCTCAGG |
|  | RP | GATGTAGGCGGAGAGGTCGAT |
| *Ppar-γ* | FP | GGAAGACCACTCGCATTCCTT |
|  | RP | GTAATCAGCAACCATTGGGTCA |
| *Gata1* | FP | TGTCCTCACCATCAGATTCCA |
|  | RP | TCCCTCCATACTGTTGAGCAG |
| *Gata2* | FP | CACCCCGCCGTATTGAATG |
|  | RP | CCTGCGAGTCGAGATGGTTG |
| *Gata3* | FP | AAGCTCAGTATCCGCTGACG |
|  | RP | GTTTCCGTAGTAGGACGGGAC |
| *Epor* | FP | GCTCCGGGATGGACTTCA |
|  | RP | GAGCCTGGTGCAGGCTACAT |
|  | Probe | CATACCAGCTCGAGGGTGAGTCACGAAAG |
| *S16* | FP | GATCGAGCCGCGCACG |
|  | RP | CAAATCGCTCCTTGCCCA |
|  | Probe | CTGCAGTACAAGTTACTGGAGCCTGTTTTGCT |

**SUPPLEMENTARY FIGURES**

**Supplementary Figure 1**

**
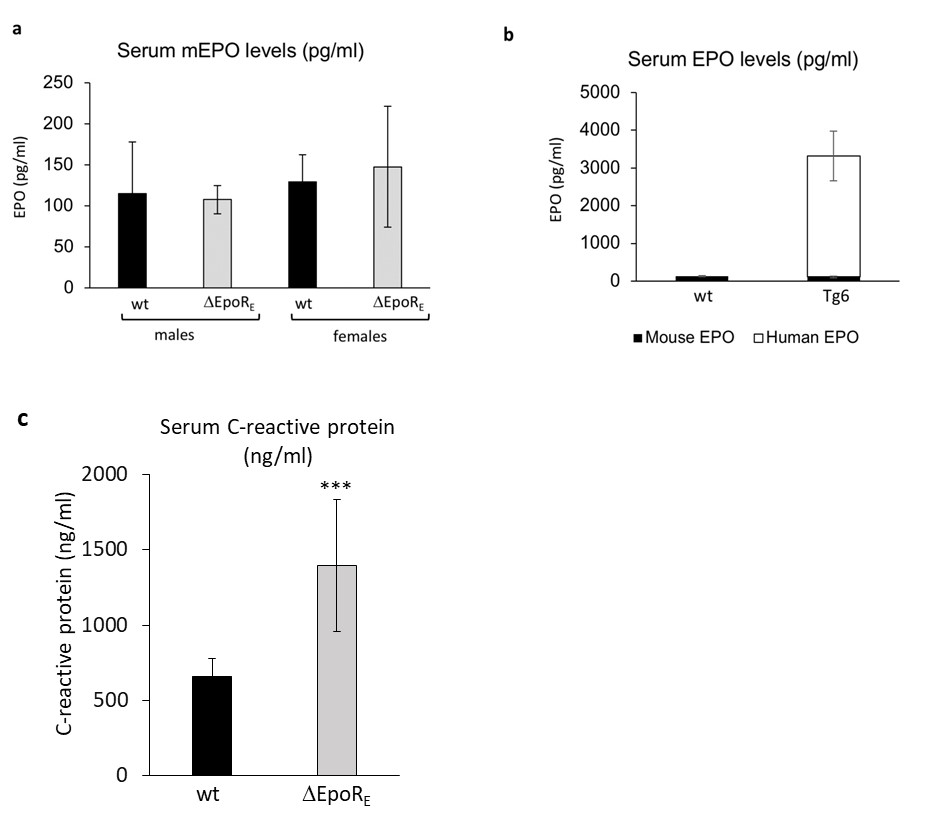
**

**Suppl Figure 1:** Serum EPO and C-reactive protein levels. **(a)** Endogenous circulating mouse EPO levels were determined from serum of wild type (wt) and ΔEpoR_E_ mice by ELISA assay. (n=5 for male-wt, female-wt and male-∆EpoR_E_-mice; n=6 for female ∆EpoR_E_-mice). (**b)** Endogenous circulating mouse EPO levels and transgenic circulating human EPO levels were determined from serum of wild type (wt) and Tg6 mice by ELISA assay specific for mouse and human EPO respectively. (n=5 for female-wt; n=4 for female Tg6-mice). No human EPO is detected in wt mice. **(c)** Levels of C-reactive protein in the serum of wild type (wt) and ΔEpoR_E_ mice quantitated by ELISA assay. (n=7 for wt, n=6 for ΔEpoR_E_ mice).

**Supplementary Figure 2**


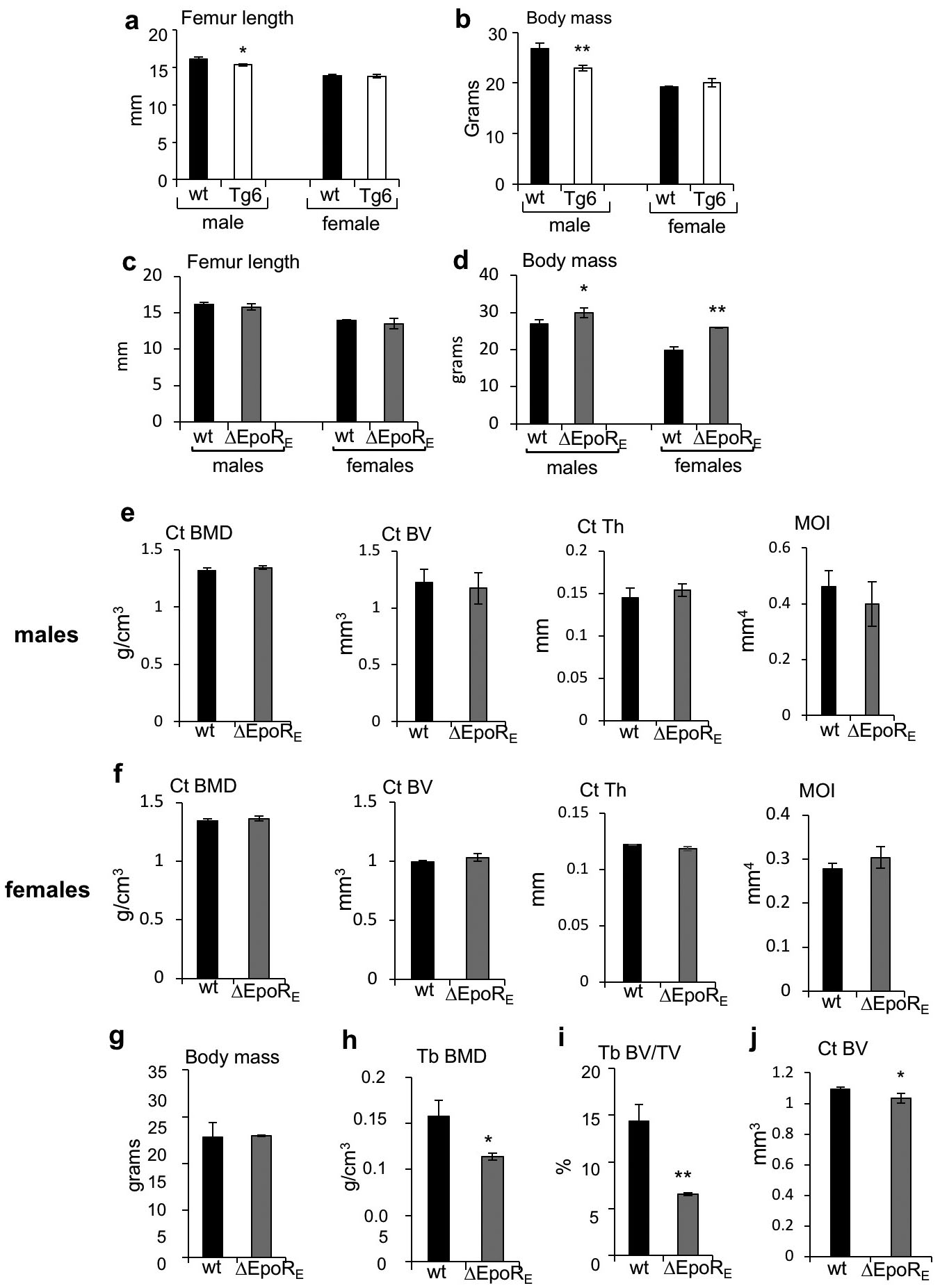


**Suppl Figure 2:** Bone characteristics and body mass of male and female Tg6 and ΔEpoR_E_ mice. (**a**) Length of the femurs of 11 week old male and female Tg6 mice and littermate controls determined by micro-Ct. **(b):** Corresponding body mass of male and female Tg6 mice and littermate controls. **(c)** Length of femurs of male and female ΔEpoR_E_ mice and wt control mice. **(d)** Body mass of male and female ΔEpoR_E_ mice and wt control mice (n=4/group). **(e-f)** Cortical bone morphometry measurements of 11 week old male (e) and female (f) ΔEpoR_E_ mice determined by micro-Ct (n=4/group, *p<0.05, **p<0.01, ***p<0.001). **(g-j)** Bone morphometry measurements of female age and weight-matched wild type C57BL6/J compared with 12-week-old ΔEpoR_E_ female mice including body mass **(g)**, trabecular bone mineral density **(h)**, trabecular bone volume **(i)** and cortical bone volume **(j)**. (n=3/group, *p<0.05, **p<0.01, ***p<0.01)

**Supplementary Figure 3**

**
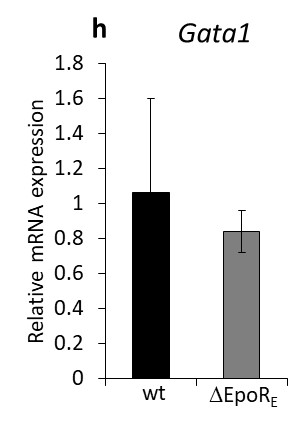
**
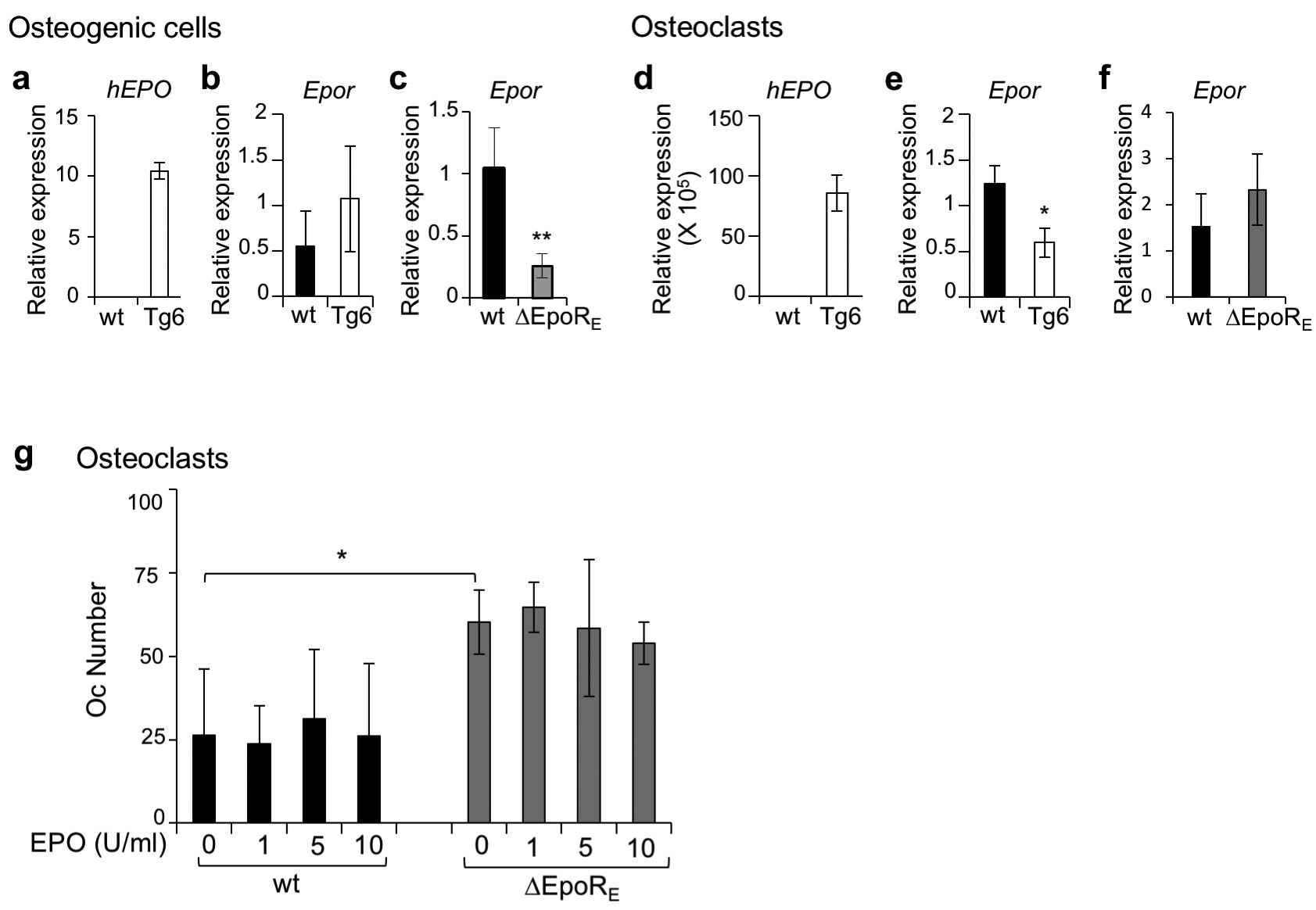


**Suppl Figure 3:** *hEPO* and *Epor* expression in osteogenic cells and osteoclasts, and osteoclast number in Tg6 and ΔEpoR_E_ mice. **(a-c**) Real-time PCR quantitation of *hEPO* **(a)** and *Epor* mRNA **(b)** in cultured wt and Tg6 osteogenic cells, and real time PCR quantification of *Epor* in wt and ΔEpoR_E_ osteogenic cells **(c)**. **(d-f)** Real-time PCR quantitation of *hEPO* **(d)** and *Epor* mRNA **(e)** in cultured osteoclasts on day 4 of wt and Tg6 osteoclasts, and real time PCR quantification of EPOR in wt and ΔEpoR_E_ osteoclasts **(f)**. (n=3-4/group, *p<0.05, **p<0.01). **(g)** Number of osteoclasts determined by TRAP staining in *in vitro* cultures of wild type and ΔEpoR_E_ osteoclast cultures treated with recombinant hEPO (1 U/ml, 5 U/ml and 10 U/ml EPO). **(h)** Real-time PCR quantitation of *Gata1* mRNA in FACS sorted wt and ΔEpoR_E_ pre-osteoclasts.

**Supplementary Figure 4**


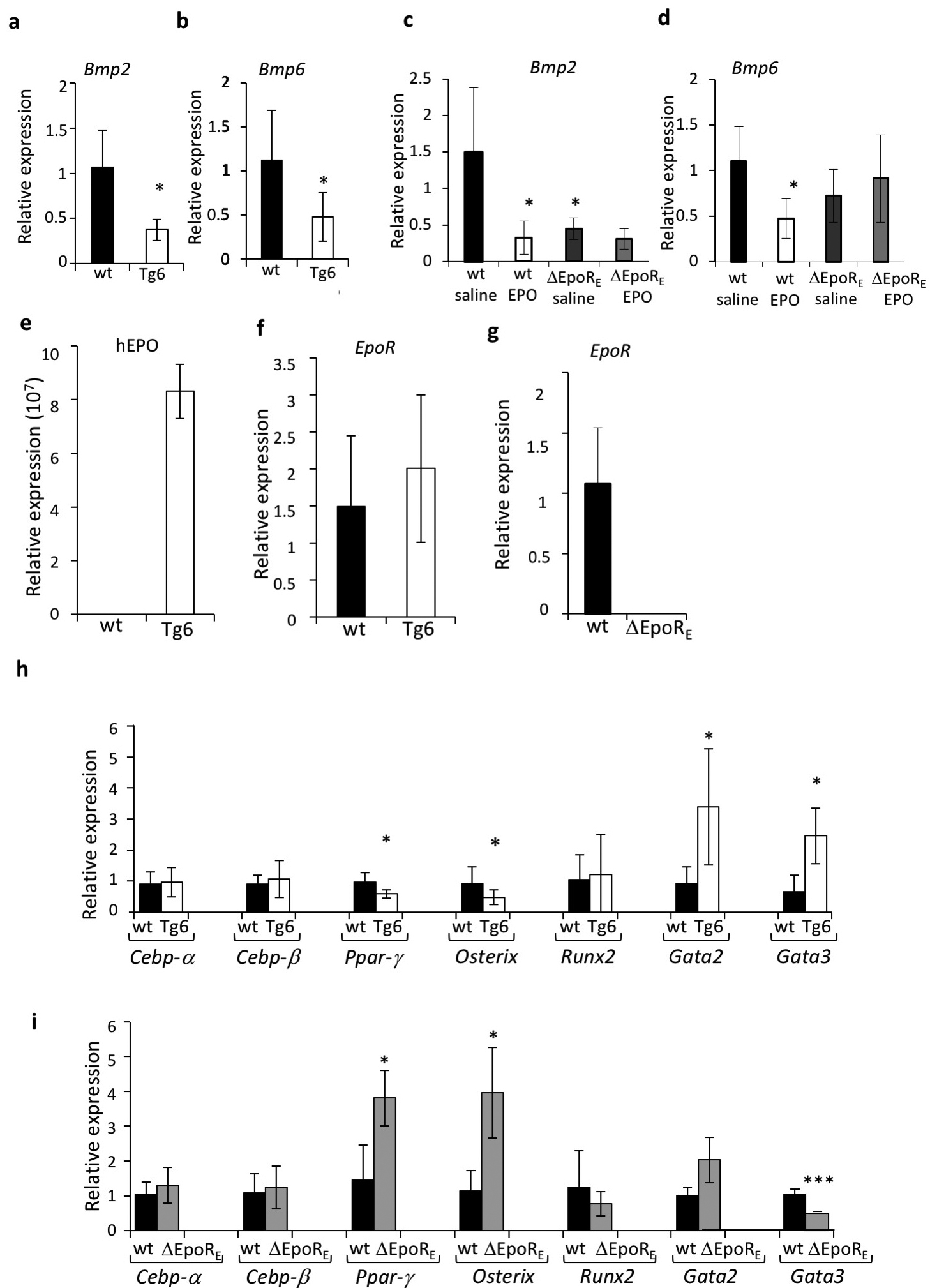


**Suppl Figure 4:** *Bmp* expression in bone marrow with EPO treatment and BMSC gene expression profile in Tg6 and ΔEpoR_E_ mice. **(a-b)** Real-time PCR quantitation of relative *Bmp2* **(a)** and *Bmp6* **(b)** mRNA in the whole bone marrow of wt and Tg6 mice. **(c-d)**: Real-time PCR quantitation of expression of *Bmp2* **(c)** and *Bmp6* **(d)** levels in the whole bone marrow of wt and ΔEpoR_E_ mice treated with 1200U EPO/kg or saline for ten days. **(e-f)** Quantitation of *hEPO* **(e)** and *Epor* mRNA **(f)** levels by PCR in wt and Tg6 BMSCs. **(g)** *Epor* expression in wt and ΔEpoR_E_ BMSCs. **(h-i)**: Quantification of transcription factors important in adipogenesis and osteogenesis in BMSCs isolated from wt littermate and Tg6 mice **(h)** and wt and ΔEpoR_E_ mice **(i)**. (n=4-5/group, p<0.05).

**Supplementary Figure 5**

**
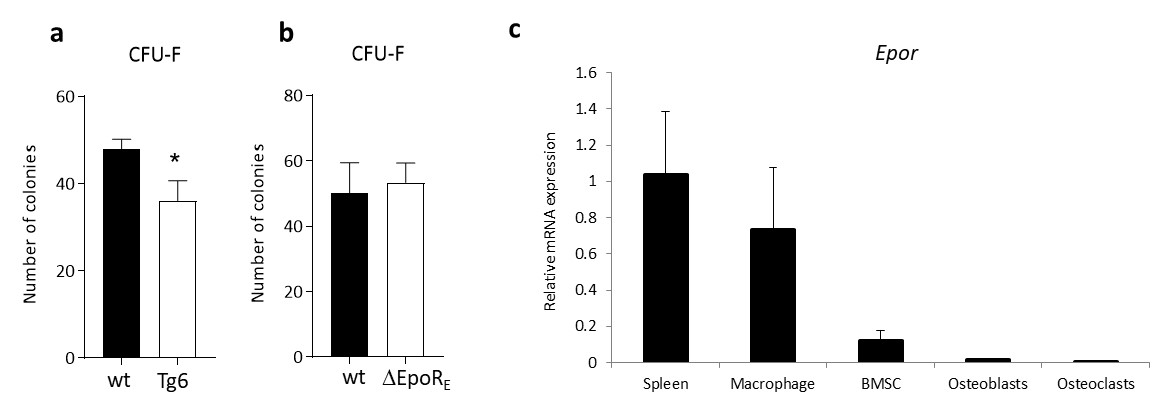
**

**Suppl Figure 5:** BMSC colony forming efficiency in Tg6 and ∆EpoR_E_ mice, and *Epor* expression in wt mice. **(a-b)** Colony forming efficiency of BMSCs was determined from the whole bone marrow using colony forming assay. **(a)** Number of colonies formed by wt and Tg6 mice (n=6/group) **(b)** Number of colonies formed by wt and ∆EpoR_E_ mice (n=5/group). **(c)** Real-time PCR quantitation of *Epor* mRNA expression in bone marrow macrophages, BMSCs, osteoblasts and osteoclasts relative to spleen.
