# Supplementary material for: Erythropoietin modulates bone marrow stromal cell differentiation
Source: Bone Res. 2019 Jul 25;7:21. doi: 10.1038/s41413-019-0060-0 (PMC6804931; doi:10.1038/s41413-019-0060-0)
Supplement: Supplementary file 2 — Supplementary Table 1 [file 41413_2019_60_MOESM2_ESM.docx]

**Supplemental table 1. Primer and probe sequences**

| **Primers and probes** |  | **Sequences** |
| --- | --- | --- |
| *Alkaline phosphatase* | FP | TTGTGCCAGAGAAAGAGAGAGA |
|  | RP | GTTTCAGGGCATTTTTCAAGGT |
| *Runx2* | FP | ACGAAAAATTAACGCCAGTCG |
|  | RP | TCGGTCTGACGACGCTAAAG |
| *OsteriX* | FP | GAGGAAGAAGCTCACTATGGCTCCAG |
|  | RP | GCCTCCTTTCCCCAGGGTTGTTGA |
| *Bmp2* | FP | CACACAGGGACACACCAACC |
|  | RP | CAAAGACCTGCTAATCCTCAC |
| *Bmp6* | FP | AGCACAGAGACTCTGACCTATTTTTG |
|  | RP | CCACAGATTGCTAGTTGCTGTGA |
| *Cathepsin K* | FP | TAGCCACGCTTCCTATCCGA |
|  | RP | TCCTCCGGAGACAGAGCAAA |
| *β-actin* | FP | GCAGGAGTACGATGAGTCCG |
|  | RP | ACGCAGCTCAGTAACAGTCC |
| *human EPO* | FP | GGAGGCCGAGAATATCACGAC |
|  | RP | CCCTGCCAGACTTCTACGG |
| *Cebp-α* | FP | GCGGGAACGCAACAACATC |
|  | RP | GTCACTGGTCAACTCCAGCAC |
| *Cebp-β* | FP | GCAGCCACTTGAGTTCTCAGG |
|  | RP | GATGTAGGCGGAGAGGTCGAT |
| *Ppar-γ* | FP | GGAAGACCACTCGCATTCCTT |
|  | RP | GTAATCAGCAACCATTGGGTCA |
| *Gata1* | FP | TGTCCTCACCATCAGATTCCA |
|  | RP | TCCCTCCATACTGTTGAGCAG |
| *Gata2* | FP | CACCCCGCCGTATTGAATG |
|  | RP | CCTGCGAGTCGAGATGGTTG |
| *Gata3* | FP | AAGCTCAGTATCCGCTGACG |
|  | RP | GTTTCCGTAGTAGGACGGGAC |
| *Epor* | FP | GCTCCGGGATGGACTTCA |
|  | RP | GAGCCTGGTGCAGGCTACAT |
|  | Probe | CATACCAGCTCGAGGGTGAGTCACGAAAG |
| *S16* | FP | GATCGAGCCGCGCACG |
|  | RP | CAAATCGCTCCTTGCCCA |
|  | Probe | CTGCAGTACAAGTTACTGGAGCCTGTTTTGCT |
