# Supplementary material for: Erythropoietin modulates bone marrow stromal cell differentiation
Source: Bone Res. 2019 Jul 25;7:21. doi: 10.1038/s41413-019-0060-0 (PMC6804931; doi:10.1038/s41413-019-0060-0)
Supplement: Supplementary file 3 — Supplementary Figure 1 [file 41413_2019_60_MOESM3_ESM.docx]

**Supplementary Figure 1**

**
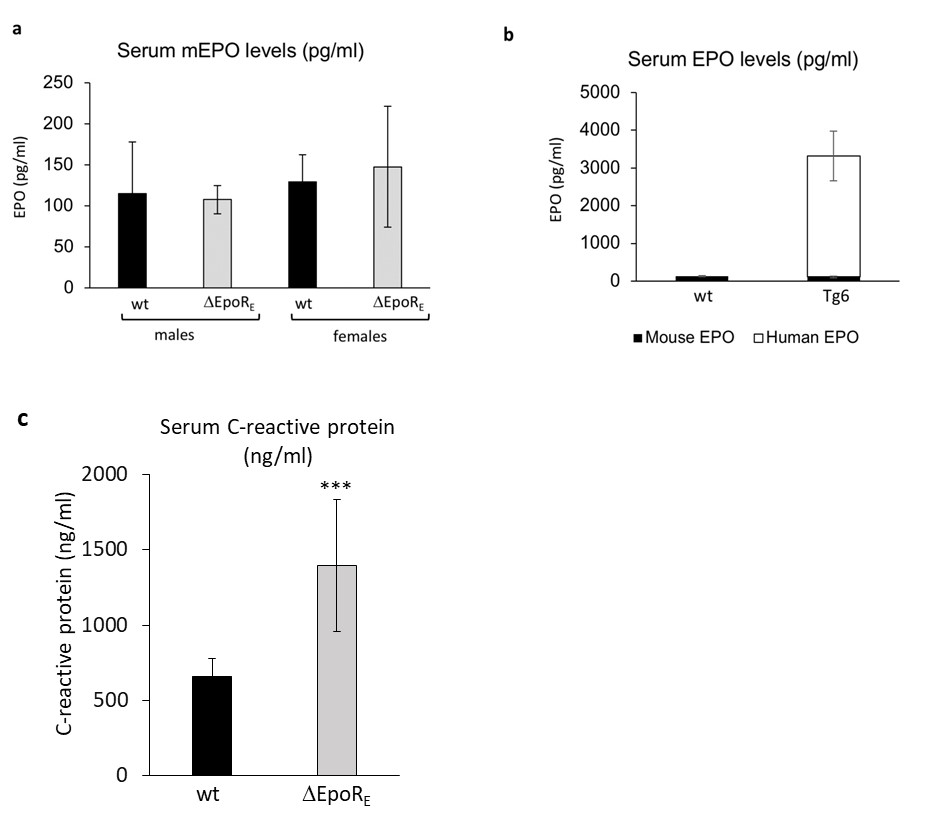
**

**Suppl Figure 1:** Serum EPO and C-reactive protein levels. **(a)** Endogenous circulating mouse EPO levels were determined from serum of wild type (wt) and ΔEpoR_E_ mice by ELISA assay. (n=5 for male-wt, female-wt and male-∆EpoR_E_-mice; n=6 for female ∆EpoR_E_-mice). (**b)** Endogenous circulating mouse EPO levels and transgenic circulating human EPO levels were determined from serum of wild type (wt) and Tg6 mice by ELISA assay specific for mouse and human EPO respectively. (n=5 for female-wt; n=4 for female Tg6-mice). No human EPO is detected in wt mice. **(c)** Levels of C-reactive protein in the serum of wild type (wt) and ΔEpoR_E_ mice quantitated by ELISA assay. (n=7 for wt, n=6 for ΔEpoR_E_ mice).
