# Supplementary material for: Erythropoietin modulates bone marrow stromal cell differentiation
Source: Bone Res. 2019 Jul 25;7:21. doi: 10.1038/s41413-019-0060-0 (PMC6804931; doi:10.1038/s41413-019-0060-0)
Supplement: Supplementary file 4 — Supplementary Figure 2 [file 41413_2019_60_MOESM4_ESM.docx]

**Supplementary Figure 2**


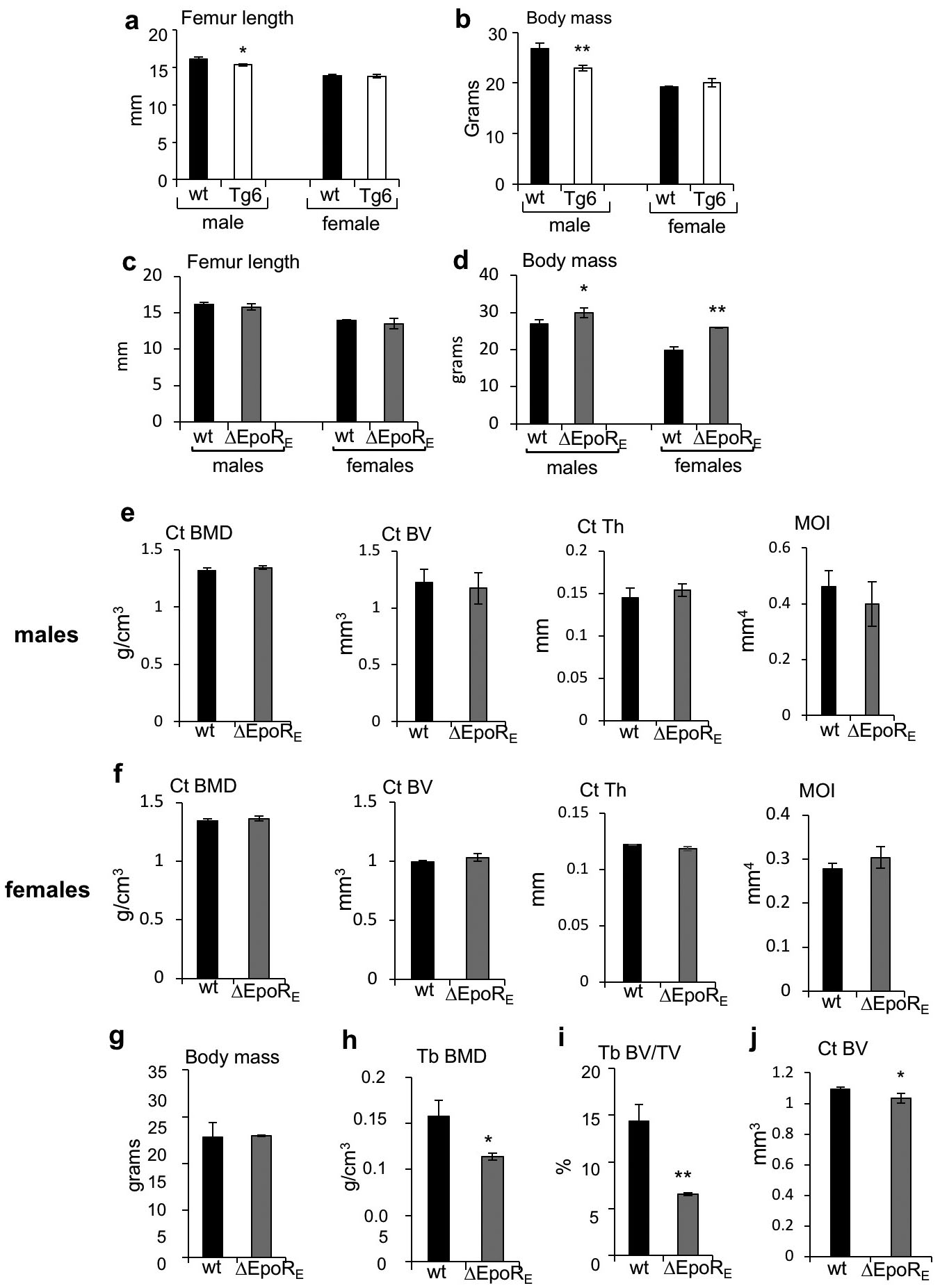


**Suppl Figure 2:** Bone characteristics and body mass of male and female Tg6 and ΔEpoR_E_ mice. (**a**) Length of the femurs of 11 week old male and female Tg6 mice and littermate controls determined by micro-Ct. **(b):** Corresponding body mass of male and female Tg6 mice and littermate controls. **(c)** Length of femurs of male and female ΔEpoR_E_ mice and wt control mice. **(d)** Body mass of male and female ΔEpoR_E_ mice and wt control mice (n=4/group). **(e-f)** Cortical bone morphometry measurements of 11 week old male (e) and female (f) ΔEpoR_E_ mice determined by micro-Ct (n=4/group, *p<0.05, **p<0.01, ***p<0.001). **(g-j)** Bone morphometry measurements of female age and weight-matched wild type C57BL6/J compared with 12-week-old ΔEpoR_E_ female mice including body mass **(g)**, trabecular bone mineral density **(h)**, trabecular bone volume **(i)** and cortical bone volume **(j)**. (n=3/group, *p<0.05, **p<0.01, ***p<0.01)
