# Supplementary material for: Erythropoietin modulates bone marrow stromal cell differentiation
Source: Bone Res. 2019 Jul 25;7:21. doi: 10.1038/s41413-019-0060-0 (PMC6804931; doi:10.1038/s41413-019-0060-0)
Supplement: Supplementary file 5 — Supplementary Figure 3 [file 41413_2019_60_MOESM5_ESM.docx]

**Supplementary Figure 3**

**
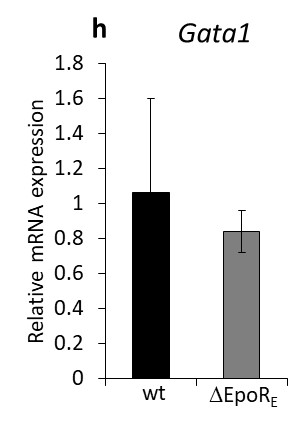
**
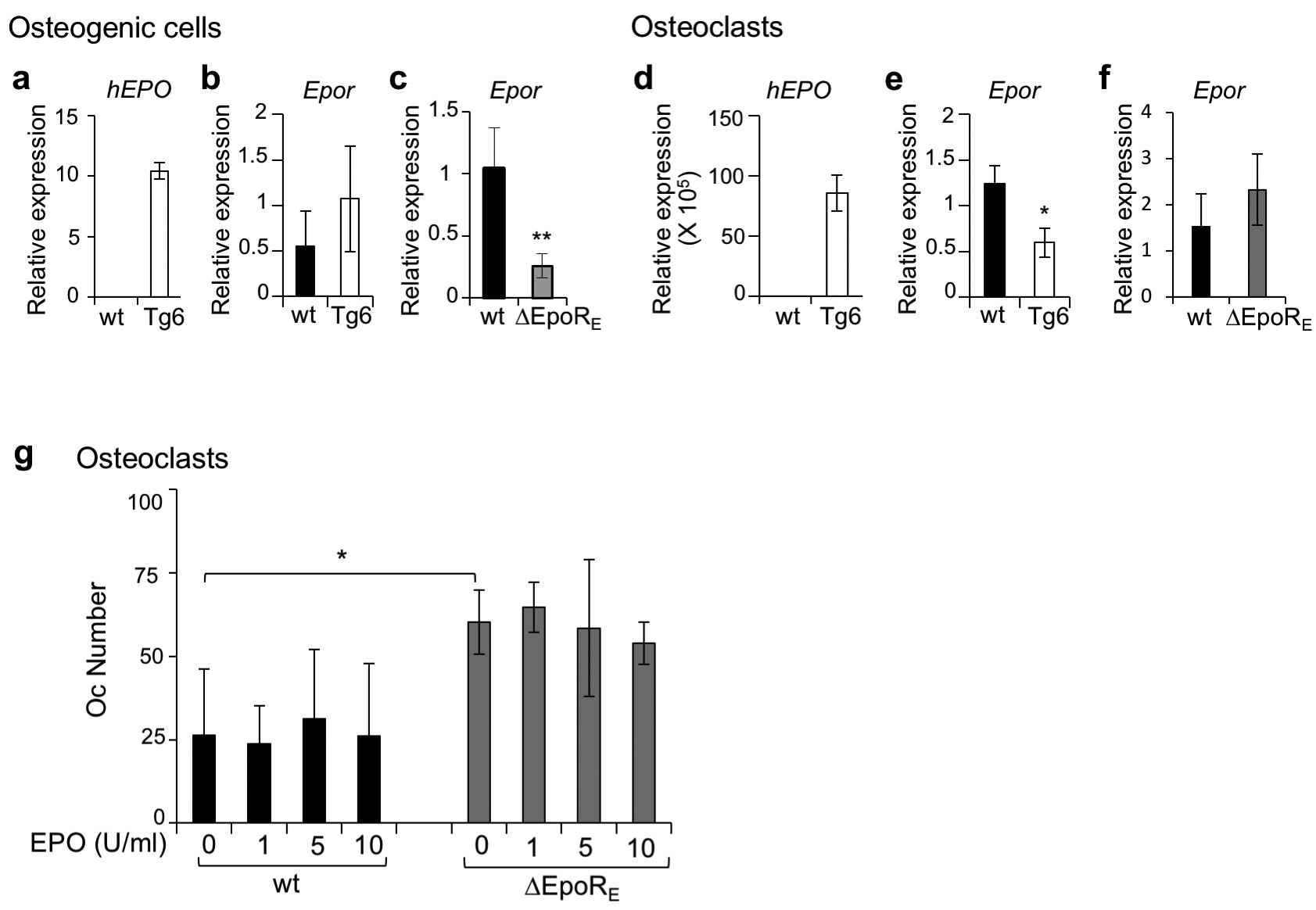


**Suppl Figure 3:** *hEPO* and *Epor* expression in osteogenic cells and osteoclasts, and osteoclast number in Tg6 and ΔEpoR_E_ mice. **(a-c**) Real-time PCR quantitation of *hEPO* **(a)** and *Epor* mRNA **(b)** in cultured wt and Tg6 osteogenic cells, and real time PCR quantification of *Epor* in wt and ΔEpoR_E_ osteogenic cells **(c)**. **(d-f)** Real-time PCR quantitation of *hEPO* **(d)** and *Epor* mRNA **(e)** in cultured osteoclasts on day 4 of wt and Tg6 osteoclasts, and real time PCR quantification of EPOR in wt and ΔEpoR_E_ osteoclasts **(f)**. (n=3-4/group, *p<0.05, **p<0.01). **(g)** Number of osteoclasts determined by TRAP staining in *in vitro* cultures of wild type and ΔEpoR_E_ osteoclast cultures treated with recombinant hEPO (1 U/ml, 5 U/ml and 10 U/ml EPO). **(h)** Real-time PCR quantitation of *Gata1* mRNA in FACS sorted wt and ΔEpoR_E_ pre-osteoclasts.
