# Supplementary material for: Erythropoietin modulates bone marrow stromal cell differentiation
Source: Bone Res. 2019 Jul 25;7:21. doi: 10.1038/s41413-019-0060-0 (PMC6804931; doi:10.1038/s41413-019-0060-0)
Supplement: Supplementary file 6 — Supplementary Figure 4 [file 41413_2019_60_MOESM6_ESM.docx]

**Supplementary Figure 4**


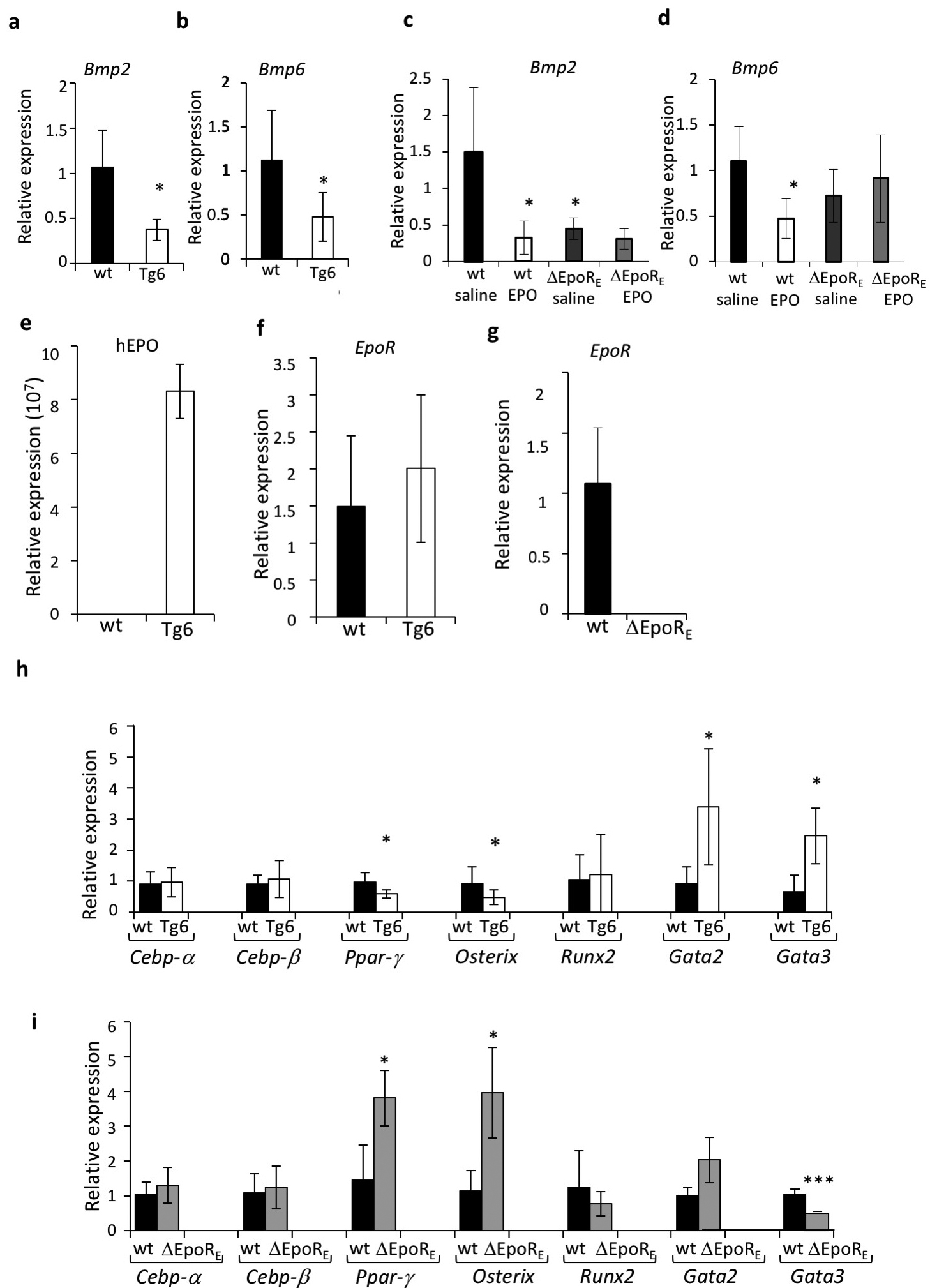


**Suppl Figure 4:** *Bmp* expression in bone marrow with EPO treatment and BMSC gene expression profile in Tg6 and ΔEpoR_E_ mice. **(a-b)** Real-time PCR quantitation of relative *Bmp2* **(a)** and *Bmp6* **(b)** mRNA in the whole bone marrow of wt and Tg6 mice. **(c-d)**: Real-time PCR quantitation of expression of *Bmp2* **(c)** and *Bmp6* **(d)** levels in the whole bone marrow of wt and ΔEpoR_E_ mice treated with 1200U EPO/kg or saline for ten days. **(e-f)** Quantitation of *hEPO* **(e)** and *Epor* mRNA **(f)** levels by PCR in wt and Tg6 BMSCs. **(g)** *Epor* expression in wt and ΔEpoR_E_ BMSCs. **(h-i)**: Quantification of transcription factors important in adipogenesis and osteogenesis in BMSCs isolated from wt littermate and Tg6 mice **(h)** and wt and ΔEpoR_E_ mice **(i)**. (n=4-5/group, p<0.05).
