# Supplementary material for: Erythropoietin modulates bone marrow stromal cell differentiation
Source: Bone Res. 2019 Jul 25;7:21. doi: 10.1038/s41413-019-0060-0 (PMC6804931; doi:10.1038/s41413-019-0060-0)
Supplement: Supplementary file 7 — Supplementary Figure 5 [file 41413_2019_60_MOESM7_ESM.docx]

**Supplementary Figure 5**

**
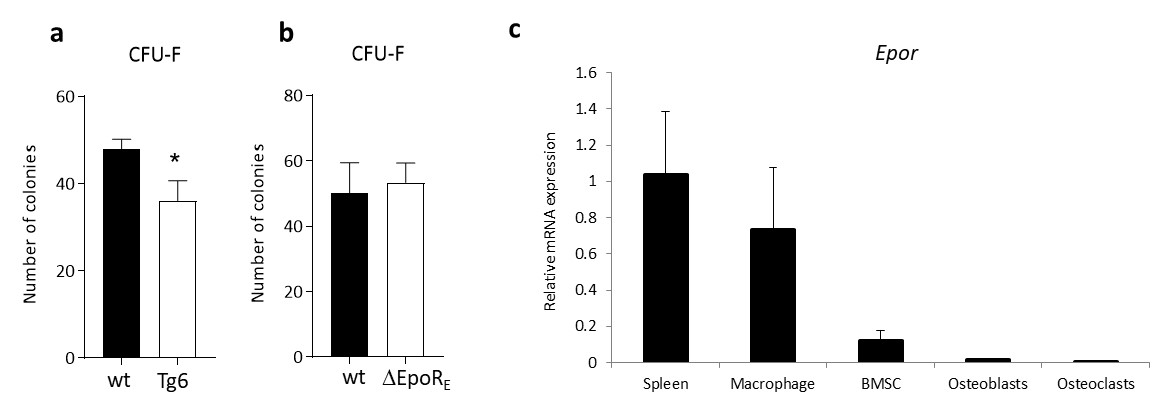
**

**Suppl Figure 5:** BMSC colony forming efficiency in Tg6 and ∆EpoR_E_ mice, and *Epor* expression in wt mice. **(a-b)** Colony forming efficiency of BMSCs was determined from the whole bone marrow using colony forming assay. **(a)** Number of colonies formed by wt and Tg6 mice (n=6/group) **(b)** Number of colonies formed by wt and ∆EpoR_E_ mice (n=5/group). **(c)** Real-time PCR quantitation of *Epor* mRNA expression in bone marrow macrophages, BMSCs, osteoblasts and osteoclasts relative to spleen.
